# Towards standardisation of cell-free DNA measurement in plasma: controls for extraction efficiency, fragment size bias and quantification

**DOI:** 10.1007/s00216-014-7835-3

**Published:** 2014-05-24

**Authors:** Alison S. Devonshire, Alexandra S. Whale, Alice Gutteridge, Gerwyn Jones, Simon Cowen, Carole A. Foy, Jim F. Huggett

**Affiliations:** 1 Molecular and Cell Biology Team, LGC Ltd, Queen’s Road, Teddington, TW11 0LY UK; 2 Statistics Team, LGC Ltd, Queen’s Road, Teddington, TW11 0LY UK

**Keywords:** Cell-free DNA, Circulating nucleic acids, Clinical/biomedical analysis, Diagnostics, Liquid biopsy, Reference materials

## Abstract

Circulating cell-free DNA (cfDNA) is becoming an important clinical analyte for prenatal testing, cancer diagnosis and cancer monitoring. The extraction stage is critical in ensuring clinical sensitivity of analytical methods measuring minority nucleic acid fractions, such as foetal-derived sequences in predominantly maternal cfDNA. Consequently, quality controls are required for measurement of extraction efficiency, fragment size bias and yield for validation of cfDNA methods. We evaluated the utility of an external DNA spike for monitoring these parameters in a study comparing three specific cfDNA extraction methods [QIAamp® circulating nucleic acid (CNA) kit, NucleoSpin® Plasma XS (NS) kit and FitAmp™ plasma/serum DNA isolation (FA) kit] with the commonly used QIAamp DNA blood mini (DBM) kit. We found that the extraction efficiencies of the kits ranked in the order CNA kit > DBM kit > NS kit > FA kit, and the CNA and NS kits gave a better representation of smaller DNA fragments in the extract than the DBM kit. We investigated means of improved reporting of cfDNA yield by comparing quantitative PCR measurements of seven different reference gene assays in plasma samples and validating these with digital PCR. We noted that the cfDNA quantities based on measurement of some target genes (e.g. *TERT*) were, on average, more than twofold higher than those of other assays (e.g. *ERV3*). We conclude that analysis and averaging of multiple reference genes using a GeNorm approach gives a more reliable estimate of total cfDNA quantity.

**Electronic supplementary material:**

The online version of this article (doi:10.1007/s00216-014-7835-3) contains supplementary material, which is available to authorized users.

**Figure:**
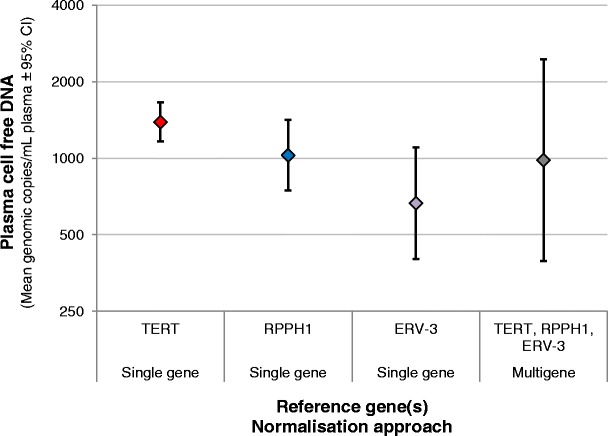
Comparison of single and multiple reference gene normalisation for quantification of plasma cell free DNA

## Introduction

The discovery of cell-free DNA (cfDNA) in blood has provided an accessible source of genetic material from foetal tissue [1], solid tumours [2, 3] or solid organ transplants [4]. Owing to the relative ease of access and the minimally invasive nature of the sampling, cfDNA could be used to simplify the longitudinal monitoring of disease progression or response to treatment in cancer patients [5], where the term ‘liquid biopsy’ has emerged to describe blood-based monitoring of tumour genetics [6]. Analysis of cfDNA also provides a safer alternative to amniocentesis or chorionic villus sampling for prenatal diagnostics [7], or to organ biopsy for the detection of transplant rejection [8]. Currently, measuring somatic mutations, such as those observed in the Kirsten rat sarcoma viral oncogene homologue gene (*KRAS*) [9], and copy number variations, such as human epidermal growth factor receptor 2 (*ERBB2*, formerly known as *HER2*) amplification in breast cancer [10] or foetal aneuploidy [11] in cfDNA, show considerable clinical potential.

One of the main hindrances in using cfDNA as a robust analyte is a lack of standardisation and appropriate controls in this field as highlighted by a number of comprehensive reports [6, 12–14]. These reviews attribute a lack of comparability between results to differences in sample processing methods and storage conditions, and in techniques for both the extraction and quantification. Recently, progress has been made in better definition of factors influencing preanalytical sample processing and storage [15–17], but variability between extraction methods and quantification approaches still presents major potential sources of experimental error [18]. In our study, we therefore focussed on the latter two aspects of cfDNA analysis.

Circulating cfDNA is a challenging analyte for extraction owing to its low concentration in plasma in normal individuals (in the region of 1.8–44 ng/mL), although this can be increased greatly in diseased or pregnant individuals [12]. Many laboratories have evaluated and compared a number of different extraction methods for the isolation of cfDNA from plasma and have demonstrated that the extraction can differ considerably in terms of efficiency depending on the method [18–26]. Historically, many of the extraction methods used in cfDNA studies, such as the QIAamp® DNA blood mini (DBM) kit and the QIAamp DSP virus kit (both from QIAGEN), were developed initially to extract high-integrity genomic DNA from blood cells or virions, and not for highly fragmented cfDNA [20, 25, 27, 28]. This could be responsible for the noted inefficiency in some extraction methods published. Although cfDNA is fragmented by nature and is present in normal individuals, tumour-derived cfDNA can differ in fragment size profile depending on the cellular process causing its release into the circulation [29, 30]. Furthermore, fetal-derived cfDNA is often more fragmented than maternal cfDNA, requiring separation approaches in order to improve recovery of the fetal-derived fraction [31].

In addition to characterisation of extraction recovery, quantification of the total amount of cfDNA is important in order to define what fraction of the total is composed of the tumour- or fetal-derived DNA. This fraction is required in non-invasive prenatal diagnosis for the calculation of the relative chromosome dosage for assessment of fetal aneuploidies [32] or relative mutation dosage approaches for single-gene disorders such as sickle cell anaemia [33], as well as tumour diagnostics for monitoring of cancer-associated copy number variations [10]. The low concentration of cfDNA extracts makes quantification of yield using methods such as UV spectroscopy or fluorescence spectroscopy problematic [34]. Assays based on quantitative PCR (qPCR) are often used for measurements of cfDNA [12]. However, qPCR also has increased variability at lower copy numbers [35], which may lead to further imprecision and bias being introduced at the quantification stage.

The emergence of kits onto the market which are specifically developed for cfDNA isolation may aid in the robustness and reproducibility of cfDNA extractions. These kits include, but are not restricted to, the QIAamp circulating nucleic acid (CNA) kit (QIAGEN), the NucleoSpin® Plasma XS (NS) kit (Macherey-Nagel) and the FitAmp™ plasma/serum DNA isolation (FA) kit (Epigentek). We present a comparison study of three specific cfDNA extraction methods with the established DBM kit. To monitor the extraction efficiency, linearity of the extraction yield, presence of co-purified inhibitors and bias associated with fragment size, we developed an in-house artificial spike-in material containing small (approximately 100 bp), medium (approximately 500 bp) and large (approximately 1,500 bp) fragment sizes. Finally, we investigate how reliable the measurement of endogenous genes is for quantification of total cfDNA in terms of bias and precision by comparing measurements of seven genomic loci in 17 individual plasma samples by qPCR and digital PCR (dPCR).

## Materials and methods

### Fragmentation of *ADH* plasmid

The pSP64 poly(A) plasmid containing the *Arabidopsis thaliana* alcohol dehydrogenase gene (*ADH*) fragment (GenBank ID M12196) was linearised with *Bgl*I as described previously [36]. The 4.5-kb linearised plasmid was fragmented in a double digest containing *Alw*NI, *Bsr*DI and bovine serum albumin to give six fragments of various sizes (67, 115, 461, 530, 1,448, and 1,889 bp). Complete linearisation and fragmentation was confirmed using a 2100 bioanalyzer and DNA 7500 series II kit (Agilent, South Queensferry, UK) according to the manufacturer’s instructions (Fig. S1).

### Human plasma samples

Plasma samples from 17 individual donors (20 mL EDTA-K_2_-treated plasma per donor) were purchased from SeraLab and stored on arrival at −80 °C. Female donors, aged 50–59 years (median donor age of 53.1 years), were from Caucasian, black or Hispanic backgrounds (Table S1). Samples were thawed prior to pooling or DNA extraction, and residual cellular debris was removed using centrifugation at 5,000 rpm for 3 min as previously described [37]. Two pools of plasma samples were prepared: plasma pools A (i) and A (ii) were prepared by mixing 8 mL from each donor sample numbered 1–5 (*n* = 2), and plasma pool B was prepared by mixing 11 mL from each donor sample numbered 6–17 (*n* = 1) (Table S1). Sample pools were homogenised by mixing them on a SpiraMixer at 4 °C for 30 min. The fragmented *ADH* plasmid was added to a 15-mL subaliquot of plasma pool A (i) as an extraction control at 10^6^ copies per millilitre of plasma [designated as “plasma pool A (i) + *ADH*”], following which further homogenisation of the plasma subpool was performed by rotation on a SpiraMixer at 4 °C for 30 min. All plasma pools were stored in aliquots [plasma pools A (i) and A (ii), 1.1 mL (all aliquots); plasma pool B, 1 × 35 mL, 2 × 45 mL] at −80 °C.

### DNA extraction

Four DNA extraction kits were used for comparison: QIAamp CNA kit (QIAGEN), QIAamp DBM kit (QIAGEN), NS kit (Macherey-Nagel) and FA kit (Epigentek). Replicate extractions were performed with plasma pool A (i) (*n* = 6) and plasma pool A (i) + *ADH* [*n* = 4; *n* = 3 (DBM kit)]. One millilitre of plasma was processed per extraction with the CNA and DBM kits and 0.5 mL plasma was processed with the NS and FA kits in line with the manufacturers’ recommended input ranges. For the CNA kit, samples were processed according to the manufacturer’s protocol for 1-mL input volume with an elution volume of 50 μL. For the DBM kit, cfDNA was extracted from plasma as described by Fleischhacker et al. [18] in spin-column format with an elution volume of 50 μL. For the NS kit, the high-sensitivity protocol was followed, including the optional proteinase digestion stage, with an elution volume of 20 μL. For the FA kit, the standard protocol was followed with an elution volume of 18 μL. Extracts were made up to final volumes of 200 μL (CNA and DBM kits) and 100 μL (NS and FA kits) with nuclease-free water, giving a final equivalent concentration of 5 μL plasma per microlitre of extract for all samples.

To assess inhibition of qPCR by components of cfDNA extracts, the two kits with the possibility of producing the maximum concentrations of cfDNA in the eluate were selected: the CNA and NS kits. Extractions were performed with plasma pool A (ii). For the CNA kit, three extractions were performed with 5 mL plasma and an elution volume of 20 μL, followed by pooling (final concentration equivalent to 1,250 μL plasma per microlitre of extract). For the NS kit, six replicate extractions were performed with 0.6 mL plasma and an elution volume of 10 μL, followed by pooling (final concentration equivalent to 300 μL plasma per microlitre of extract). Subsequently, dilutions were prepared in nuclease-free water.

For investigation of the linearity of extraction efficiency, plasma input volumes of 1, 2, 3 and 5 mL (*n* = 3) from plasma pool B were processed with the CNA kit, which has the largest range of possible plasma input volumes of the kits tested, and were eluted in 50 μL elution buffer. Three independent sets of extractions were performed. Extracts were analysed undiluted by qPCR (equivalent to 20 μL plasma per microlitre of extract).

Two sets of DNA extractions from 17 individual donor plasma samples were performed with 1 mL plasma using the CNA kit with elution volumes of 50 μL (Fig. 5) and 20 μL (Figs. 6, 7). The smaller final volume was used to increase the cfDNA concentrations in extracts for dPCR analysis. For qPCR analysis, extracts were diluted to the equivalent 25 μL plasma per microlitre of extract.

### Real-time qPCR

Real-time qPCR assays for human genomic targets telomerase reverse transcriptase (*TERT*) and ribonuclease P RNA component H1 (*RPPH1*) were designed using Primer Express® (Applied Biosystems). Additional assays for human genomic targets *ALUJ* [38], endogenous retrovirus group 3 (*ERV3*) [28], glyceraldehyde 3-phosphate dehydrogenase (*GAPDH*) [39] and *N*-acetylglucosamine kinase (*NAGK*) [40] were based on previous publications. A commercial assay ValidPrime™(VP) designed to a non-transcribed genomic locus present at one copy per haploid normal genome was obtained from TATAA Biocenter (Göteborg, Sweden). Sequences present in the *ADH* plasmid insert sequence were quantified using *Adh*β and *Adh*δ assays [36] or an assay for the 115-bp fragment of the plasmid backbone ‘*ADH*-115 bp’ (Fig. S1) designed using Primer Express. Detailed information relating to PCR assays is provided in Table S2. All qPCR experiments were performed in accordance with the MIQE guidelines [35] (Table S3, part A).

Triplicate qPCR assays were performed for each point of the standard curve, and test samples were assessed in single reactions, unless stated otherwise in the figure legends. For the endogenous targets and *ALUJ* assays, a seven-point fivefold dilution series (from approximately 3,042 to approximately 0.2 haploid genome copies per reaction) of female human genomic DNA (Promega) prepared in yeast transfer RNA (50 ng/μL) (Sigma) diluent was used for generation of the standard curve. *ALU* is a repetitive sequence found at high copy number in the genome [41, 42], and so the standard curve was generated as genome equivalents rather than copies as for the other genomic targets. To assess recovery of the exogenous spike-in from cfDNA extractions, a four-point tenfold dilution series (from 50,000 to 50 copies per reaction) of the fragmented *ADH* plasmid (diluent, nuclease-free water) was used to measure the number of copies of each *ADH* plasmid fragment. *ADH*-115 bp, *Adh*β and *Adh*δ assays were used to measure the recovery of the 115-, 461- and 1,448-bp *ADH* plasmid fragments, respectively. Assessment of qPCR inhibition of the *Adh*β assay by cfDNA extracts using the CNA, NS and FA kits (without addition of the *ADH* plasmid before extraction) was performed using the same dilution series of *ADH* plasmid in the presence of 5 μL cfDNA extract and qPCR efficiency determined on the basis of the slope of the linear regression analysis of quantification cycle (*C*_q_) versus concentration (copies per reaction).

To further assess inhibition of qPCR by components of cfDNA extracts prepared using the CNA and NS kits, fragmented *ADH* plasmid (500 copies) was added to each *Adh*β assay in addition to 5 μL of concentrated or diluted cfDNA extract, and *C*_q_ values were compared with the control condition (absence of cfDNA extract).

For all experiments unless otherwise stated, 20-μL reactions containing 5 μL sample were performed using universal master mix or gene expression master mix (Table S2) and the 7900HT fast real-time PCR system (all Life Technologies). For all experiments, no-template controls were performed with the addition of diluent without cfDNA extract or *ADH* plasmid. Results for the no-template controls are given in Table S4. The thermal cycling conditions were as follows: 50 °C for 2 min, 95 °C for 10 min, 40 cycles of 95 °C for 15 s then 60 °C for 60 s. For the *ALUJ* assays, 20-μL reactions containing 5 μL sample were performed using Power SYBR® Green master mix (Life Technologies) using the same cycling conditions as mentioned above with the addition of a melting step at the end to check the dissociation curve for the presence of primer-dimers and non-specific products: 95 °C for 15 s, 60 °C for 15 s followed by an increase in temperature to 95 °C at a ramp rate of 2 %. SDS version 2.4 (ABI) was used to calculate *C*_q_, which is defined as the number of cycles at which the fluorescence signal is significantly above the threshold. Data were exported for further analysis in Microsoft Excel® 2003/2007.

### Droplet dPCR

The details and sequences of the dPCR assays were identical to those used in real-time qPCR experiments (Table S2). Digital PCR (dPCR) experiments using the QX100™ Droplet Digital™ PCR system (Bio-Rad) were performed in accordance with the dMIQE guidelines [43] (Table S3, part B). Final-volume reactions of 20 μL containing ddPCR™ supermix (Bio-Rad) and 3.5 μL sample were established prior to droplet formation using the QX100 droplet generator according to the manufacturer’s instructions. Briefly, each 20-μL reaction mixture was pipetted into the sample well of a DG8 cartridge with 70 μL generator oil pipetted into the oil well. Droplets were generated by the QX100 droplet generator, and 40 μL of droplets was transferred, using a multichannel pipette, into a 96-well plate. Each plate was sealed with foil using a PX1™ PCR plate sealer, and PCR was performed using a C1000 Touch™ thermal cycler. The thermal cycling conditions were as follows: 95 °C for 10 min, 40 cycles of 94 °C for 30 s then 60 °C for 30 s followed by 98 °C for 10 min and cooling to 4 °C. Droplets were analysed using the QX100 droplet reader according to the manufacturer’s instructions. Data were analysed using QuantaSoft™ version 1.3.2.0 to count the number of positive droplets (*m*) and the total number of accepted droplets (*n*). The mean number of copies per droplet (*λ*) was estimated using the relationship *λ* = − ln(1 − *m*/*n*), which assumes a Poisson distribution for the number of copies in each droplet (Table S5) [43, 44]. Data were exported for further analysis into Microsoft Excel 2003/2007. The number of template copies per microlitre was calculated using the published manufacturer’s volume of 0.91 nL per droplet, and confidence intervals were calculated as described previously [45]. For all experiments, no-template controls were established in parallel with diluent added without cfDNA extract (Table S5).

### Data analysis

Data were analysed in Microsoft Excel 2003/2007 and Graphpad Prism® version 5.04. All data were converted to the number of copies per millilitre of plasma, where one copy is either for the single gene measured or as a single human haploid genome that is calculated as 3.3 pg. Reference gene stability of seven reference genes in the group of 17 donors (Fig. 5) was analysed using the GeNorm algorithm in GenEx Enterprise version 5.3.6 (MultiD Analyses, Göteborg, Sweden) [46].

**Fig. 1:**
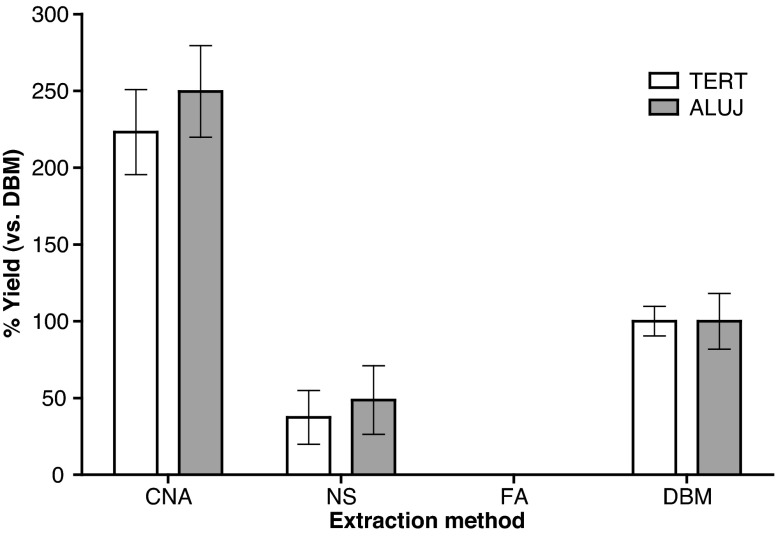
Assessment of cell-free DNA (cfDNA) yield using four extraction methods. The mean yield ± one standard deviation from replicate extractions using plasma pool A (i) (with or without *ADH*) performed with the QIAamp circulating nucleic acid (*CNA*), NucleoSpin Plasma XS (*NS*) and FitAmp plasma/serum DNA isolation (*FA*) kits (*n* = 10) and the QIAamp DNA blood mini (*DBM*) kit (*n* = 9) is displayed relative to the mean yield of the DBM kit. The yield of cfDNA was quantified by quantitative PCR (qPCR) assays to *TERT* and *ALUJ*

**Fig. 2:**
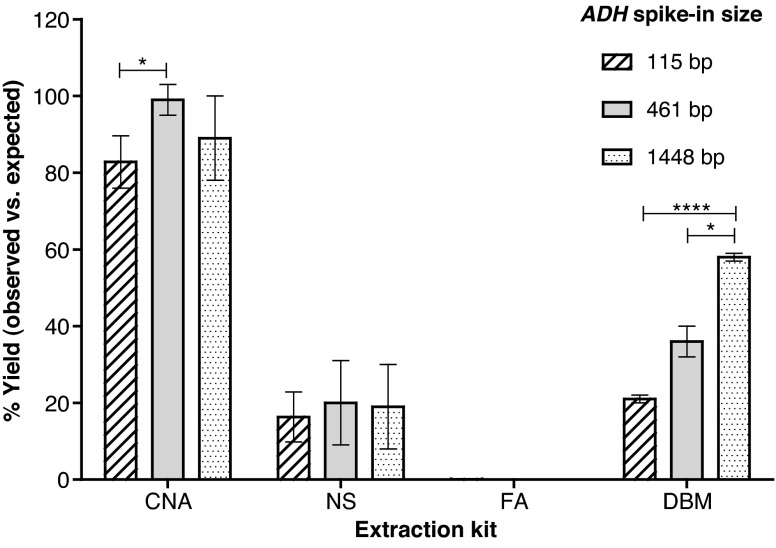
Assessment of extraction efficiency and fragment size bias of four extraction methods using an exogenous spike-in. Extraction efficiencies of the CNA, NS, FA and DBM kits are expressed as a percentage of input (10^6^ copies per millilitre of plasma) ± one standard deviation (*n* = 3 extractions for the DBM kit, *n* = 4 extractions for the other methods) for 115-, 461- and 1,448-bp fragments of the *ADH* plasmid spike-in. Significant differences between the yields of the *ADH* plasmid fragments are indicated: *one asterisk*, *p* < 0.05; *four asterisks*, *p* < 0.0001

**Fig. 3:**
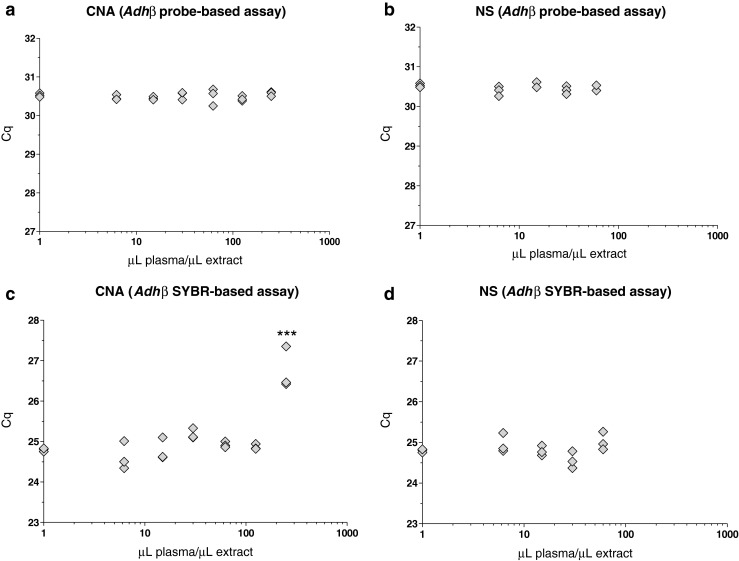
Assessment of qPCR inhibition by cfDNA extracts using two different extraction methods. Fragmented *ADH* plasmid (500 copies per reaction) was measured using the *Adh*β assay (qPCR, *n* = 3) in both hydrolysis probe (**a**, **b**) and intercalating dye (**c**, **d**) assay formats in the presence of increasing concentrations of cfDNA extract produced using the CNA (**a**, **c**) and NS (**b**, **d**) kits and compared with control conditions (given a nominal concentration of 1 μL plasma per microlitre of extract owing to plotting on a log scale). Individual data points represent replicate qPCR assays. An increase in *C*_q_ indicates inhibition of qPCR versus the control condition. *Three asterisks*, *p* < 0.001

**Fig. 4:**
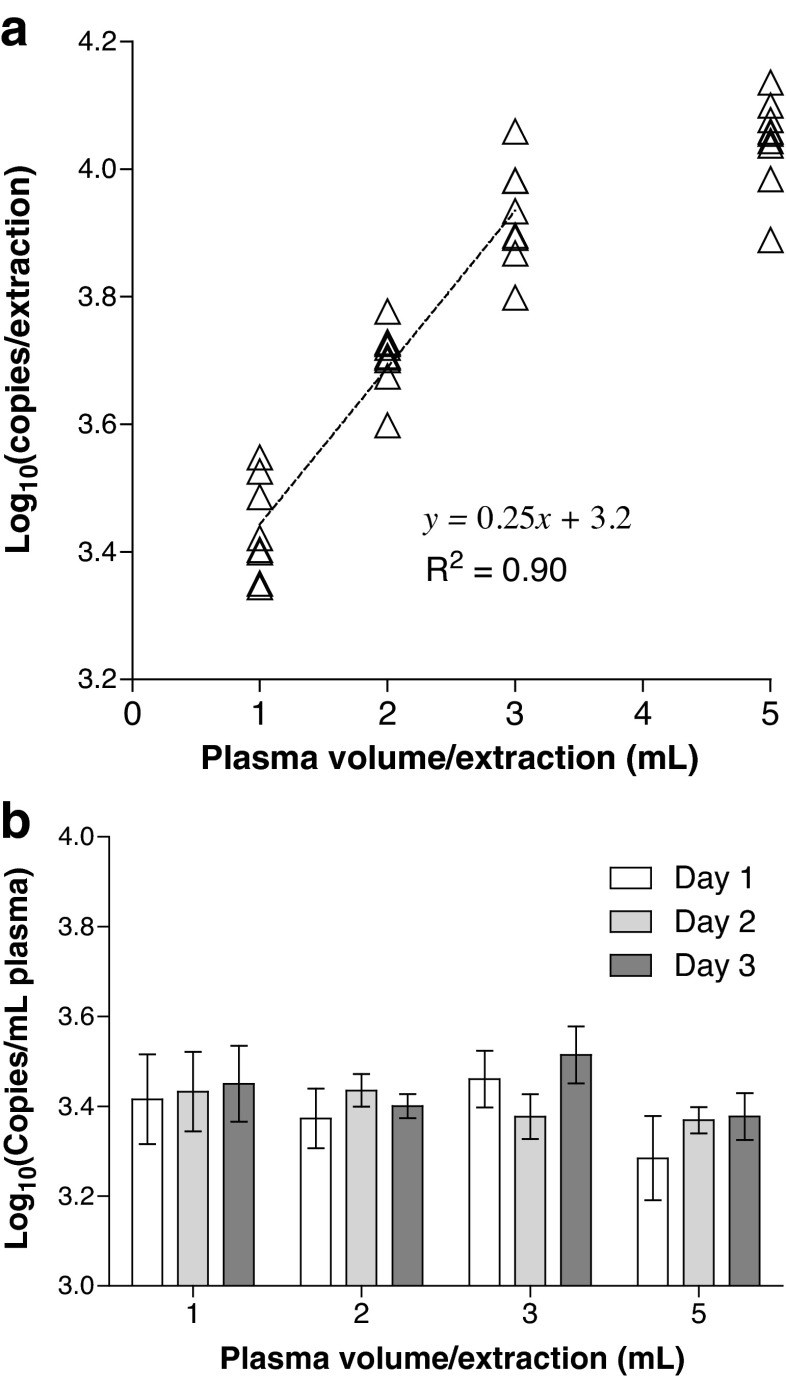
Linearity and within-laboratory reproducibility of cfDNA extraction using a single method. Three independent extraction experiments performed on different days using the CNA kit were performed with 1, 2, 3 and 5 mL plasma pool B (*n* = 3 replicates per day). **a** The yield of cfDNA (copies per extraction) is compared with the volume of plasma (mL) processed per extraction using the CNA kit (*n* = 9 per volume). Data points correspond to individual extracts. **b** The within-laboratory reproducibility of extract yield per millilitre of plasma is compared for different input volumes and three independent experiments. Mean values ± one standard deviation are plotted for each day (*n* = 3)

**Fig. 5:**
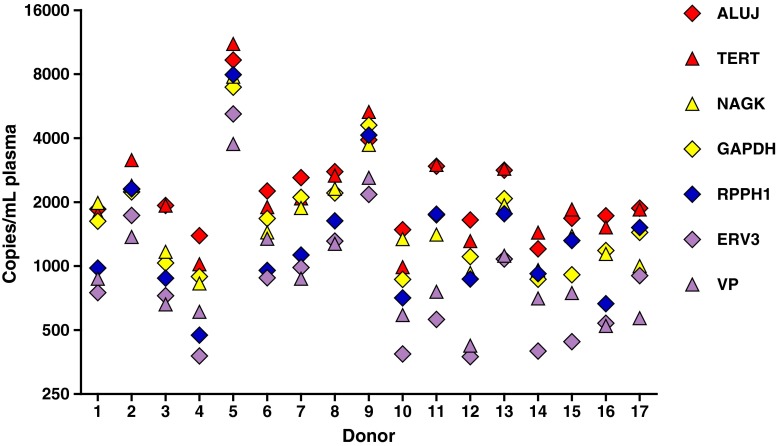
Comparison of cfDNA copy number of seven different reference genes in 17 donor samples measured by qPCR assays. The results for each reference gene are displayed with a different symbol for each target

**Fig. 6:**
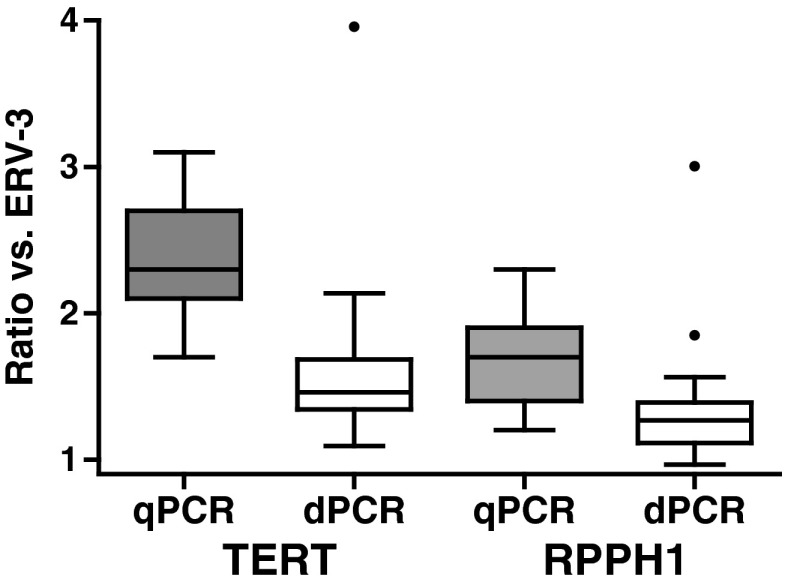
Comparison of fold differences between reference genes measured by qPCR and droplet digital PCR (*dPCR*). The ratios of *TERT*/*ERV3* and *RPPH1*/*ERV3* genomic copy numbers in extracts from 17 plasma samples are compared for qPCR-based and droplet-dPCR-based measurements. Box and whisker plots depict the median value (*line*), interquartile range (*box*) and upper and lower limits (*whiskers*) based on Tukey’s honest significant difference test. Outlier values are indicated as single points

**Fig. 7:**
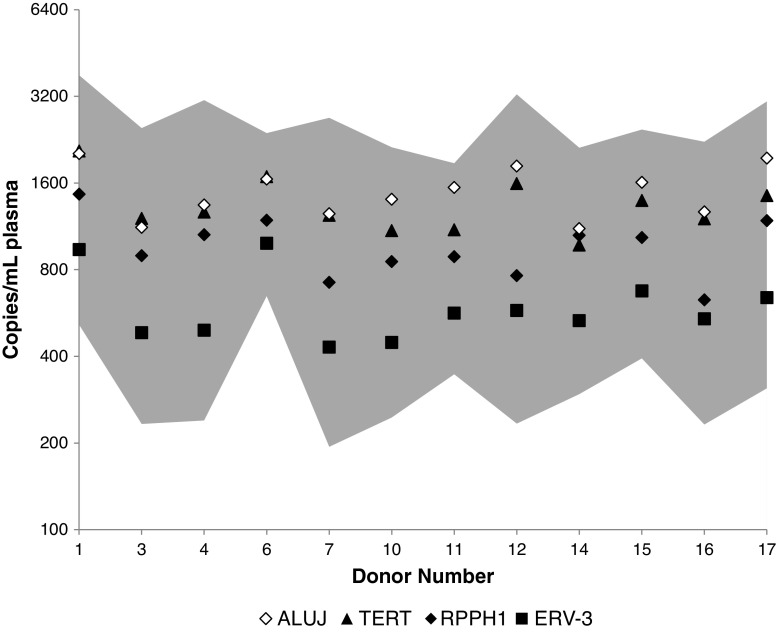
Comparison of a multiple reference gene approach for plasma cfDNA quantification with single gene measurements. The 95 % confidence interval (*grey area*) associated with a normalised geometric average cfDNA quantity calculated on the basis of three reference genes (*TERT*, *RPPH1*, *ERV3*) and three independent qPCR experiments is compared with mean estimates for each of the above-mentioned reference genes and *ALUJ* for each donor

One- and two-way analysis of variance (ANOVA) tests (Figs. 1, 2, 3, 4b), regression analysis (Fig. 4a) and Pearson correlation analysis (Table S5) were performed using Graphpad Prism version 5.04. Analysis of reference gene copy numbers in cfDNA extracts (Figs. 5, 6, 7) was performed with log_10_-transformed values in R version 3.0.1 [47] using linear models, with the reference gene and the donor as fixed variables. Two-way ANOVA was performed, and differences between reference genes were compared using Tukey’s honest significant difference test (see the electronic supplementary material). Further information is given in the electronic supplementary material.

An average cfDNA quantity for each donor based on *TERT*, *RPPH1* and *ERV3* copy numbers (GeNorm approach) was calculated as the arithmetic mean of log_10_-transformed values. We calculated 95 % confidence intervals of the GeNorm average cfDNA quantity on the basis of errors associated with the reference gene and qPCR replicate by one-way ANOVA (Graphpad Prism) (see the electronic supplementary material). The mean square between-group (MS_r_) variance was used to calculate the standard error of the mean *σ*:

1 $$ {\sigma}^2=\frac{{\mathrm{MS}}_{\mathrm{r}}}{ nb} $$

where *b* is the number of groups (reference genes) and *n* is the number of replicate qPCR measurements. The 95 % confidence interval was calculated by multiplying the standard error *σ* by the coverage factor *k* associated with two degrees of freedom (three groups) for *α* = 0.05 (*k* = 4.3). The log-transformed values were transformed to linear scale (Fig. 7).

## Results

We compared three kits specifically developed for the extraction of cfDNA from body fluids (CNA, NS and FA kits) with the DBM kit (which has been used widely for this application [18]) using plasma pool A (i). The yields of cfDNA in terms of genome equivalents per millilitre of plasma for each of the four methods were assessed by qPCR measurement of two reference assays, one for the single-copy gene *TERT*, and the other for the genomic repetitive element (*ALUJ*). The values are expressed as percent yield compared with the mean yield of the DBM kit (Fig. 1). The results were consistent between both reference assays and suggested that the yield of cfDNA per millilitre of plasma was between 2.2-fold and 2.5-fold higher with the CNA kit than with the DBM kit, whereas the NS extracts contained less than half the cfDNA yield of the DBM extracts. Extracts obtained using the FA kit contained very low levels of cfDNA, which were not detectable by the *TERT* assay and were estimated to contain approximately one genome copy per millilitre of plasma by the *ALUJ* assay (Fig. 1).

To investigate further extraction kit DNA recovery and DNA fragment bias, a spike-in containing the digested *ADH* plasmid was added to a subpool of plasma prior to isolation of cfDNA [plasma pool A (i) + *ADH*]. The recovery of the 115-, 461- and 1,448-bp fragments was measured after extraction using three qPCR assays detecting sequences present in these fragments (*ADH*-115 bp, *Adh*β and *Adh*δ, respectively) in order to evaluate the fragment size profile of the extraction methods (Fig. 2).

In line with the results from the endogenous targets (Fig. 1), the extraction efficiency of the CNA kit was the highest of the four methods, with over 80 % recovery for all three fragment sizes. Average approximately twofold and approximately 4.8-fold lower extraction efficiencies were noted for the DBM and NS methods, respectively (Fig. 2). For the FA kit, recovery of the exogenous DNA was also lower (less than 0.1 % recovery of all three *ADH* fragments). The NS kit demonstrated an even profile in terms of recovery of both smaller and larger DNA fragments. Although the CNA kit recovered a high percentage (83 %) of the smallest plasmid fragment (115 bp), the yield of this was not as high as that of the 461-bp fragment (99 %) (*p* < 0.05). The DBM kit demonstrated the highest recovery of the largest, 1,448-bp fragment (58 %), compared with 37 % recovery of the 461-bp fragment (*p* < 0.05) and 21 % recovery of the 115-bp fragment (*p* < 0.0001). The repeatability associated with replicate extractions for each kit was consistent between the results of endogenous and plasmid targets, with the CNA and DBM kits demonstrating a mean repeatability percent coefficient of variation (CV) of 10 % for all assays, compared with 49 % for the NS kit (Table S6) (data not presented for the FA kit owing to the low yield).

One advantage of the CNA and NS kits is the ability to concentrate cfDNA, through the use of either large collection tubes for processing up to 5 mL plasma (CNA kit) or a specially designed purification column allowing elution volumes as low as 5 μL (NS kit). These more concentrated extracts are ideally suited for new technologies such as dPCR, which typically use smaller volumes of samples per assay compared with qPCR [45, 48]. However, these highly concentrated extracts may also contain higher concentrations of PCR inhibitors present in the extraction matrix (such as blood tube preservatives [49]). To investigate this, replicate extractions were performed using plasma pool A (ii) with the maximum plasma input volume of the two kits (5 mL for the CNA kit and 0.6 mL for the NS kit) together with the minimum elution volumes (20 and 10 μL, respectively), followed by pooling after extraction before serial dilution to test whether the concentrated extracts inhibited qPCR (Fig. 3). Inhibitory substances in the extracts were assayed by spiking the qPCR with *ADH* plasmid and measuring *C*_q_ using the *Adh*β assay. The assay was performed in two formats with either a hydrolysis probe (Figs. 3a, b, S2) or using intercalating dye chemistry (Fig. 3c, d). An increase in *C*_q_ versus the control reaction was judged to indicate inhibition of the qPCR. Reactions contained 4 μL sample with concentrations equivalent to 6.25, 15, 30 and 60 μL plasma per microlitre for the CNA and NS kits, and additionally 125 and 250 μL plasma per microlitre in the case of the CNA kit. The TaqMan-based *Adh*β assay did not show any evidence of inhibition with extracts from either the CNA (Fig. 3a) or the NS (Fig. 3b) extracts. The CNA kit extracts did not inhibit the SYBR-based *Adh*β assay up to concentrations of 125 μL plasma per microlitre; however, at the maximum concentration of 250 μL plasma per microlitre of extract (equivalent to 1 mL plasma extract per qPCR), a mean shift of approximately 2.0 *C*_q_ units was observed (Fig. 3c). The NS kit extracts did not inhibit the SYBR-based *Adh*β assay (Fig. 3d).

On the basis of the results of the assessments of yield, fragment size distribution and presence of inhibitors (Figs. 1, 2, 3), the CNA kit was chosen as the most suitable extraction method for high-sensitivity cfDNA analysis. We further evaluated the performance of this kit in terms of linearity of yield and within-laboratory reproducibility (intermediate precision). Plasma can be processed using this kit with input volumes between 1 and 5 mL, and so we tested whether the cfDNA yield was proportional to the plasma input volume by performing independent extractions of plasma pool B with 1, 2, 3 or 5 mL plasma (*n* = 3 replicates per experiment) on three different days and with elution in a fixed volume. The yield of cfDNA was assessed using the *TERT* assay (Fig. 4). Log-transformed cfDNA yields in the CNA extracts were normally distributed (analysis not shown) and were linear with respect to input volume up to an input volume of 3 mL plasma per extraction (*R* ^2^ = 0.90) (Fig. 4a). Yield expressed as per millilitre of plasma was found to be significantly different between the four different input volumes on the basis of ANOVA (*p* = 0. 015). This is consistent with the regression analysis (Fig. 4a), and suggests a slight reduction in extraction efficiency at 5 mL plasma per extraction (Fig. 4b). However, post hoc analysis did not indicate significant differences between specific volumes (data not shown). No significant differences in yield per millilitre of plasma were found between the three independent extractions (Fig. 4b). The within-laboratory reproducibility was calculated as a CV of 17 %, composed predominantly of within-day variability (16 %), which was consistent with repeatability data using 1 mL plasma per extraction (Fig. 1, Table S6).

Having assessed different technical aspects of the performance of cfDNA extraction methods, we investigated controls for the quantification of cfDNA extraction and downstream assay normalisation to total cfDNA. The total cfDNA isolated from plasma samples from 17 individual donors was quantified using seven different reference assays. In addition to an assay for *TERT*, a number of assays for single-copy genomic targets commonly used for cfDNA measurements (*RPPH1*, *GAPDH*, *NAGK* and *ERV3*) were also used, as well as a commercial assay VP, designed for an undisclosed single-copy locus. These results were compared with the genome equivalent estimate based on measurement of the *ALUJ* repeat element.

Analysis of all seven assays demonstrated that most of the samples contained a mean of fewer than 2,500 copies per millilitre of cfDNA (equivalent to 8 ng/mL or less). However, samples from two donors (donors 5 and 9) contained larger amounts of cfDNA [mean (range) 24.6 (12.4–36.6) ng/mL and 12.5 (7.2–17.5) ng/mL, respectively] (Fig. S3). The seven assays showed strong correlation based on the donor, with correlation coefficients (*r*) between 0.86 and 0.97 (Table S7). The reference genes were also analysed in terms of stability according to the GeNorm algorithm, which is commonly applied to reference gene normalisation of messenger RNA measurements [46] (Table S8). Good stability (*M*) values were calculated for all seven assays (*M* < 0.4), suggesting all reference genes could be suitable markers of cfDNA quantity. However, absolute differences between the genomic copies were observed (Fig. 5). On the basis of linear modelling of results from all donors, copy number measurements were ranked in the following order: *TERT* ~ *ALUJ* > *NAGK* ~ *GAPDH* > *RPHH1* > *ERV3* ~ VP. *TERT*- or *ALUJ*-based copy number measurements were, on average, between 138 and 173 % greater than those based on *ERV3* or VP, whereas *RPPH1* copy numbers were approximately 60 % higher than those of *ERV3* (see the electronic supplementary material).

We speculated as to whether the discordance between individual reference gene measurements could be attributable to artefacts of the standard calibration curve. Selected samples were therefore analysed by droplet dPCR, which does not require a standard curve for absolute quantification (Fig. S4). Measurements were performed with three assays representative of high (*TERT*), medium (*RPPH1*) and low (*ERV3*) copy numbers. There was good agreement between the qPCR and droplet dPCR measurements of copy numbers (Fig. S5). When the results of the three different assays were compared by linear modelling, the same rank order of copies per millilitre of extract was observed as for qPCR, although the fold differences were not as large. For example, *TERT* and *RPHH1* copy number measurements were on average 50 % and 30 % higher than those of *ERV3* (vs 165 % and 60 %, respectively, by qPCR) (Fig. 6; see also the electronic supplementary material).

The observed differences between qPCR and droplet dPCR, within plasma samples, demonstrate how quantification biases in the total cfDNA could be introduced. Calculation of an average value based on measurement of multiple reference assays was therefore investigated as a means of improving the accuracy of the cfDNA load quantification, by comparing the cfDNA load estimated by single reference gene measurements (*TERT*, *RPHH1* and *ERV3*) with the mean of all three targets. Precision was calculated by performing three independent qPCR experiments with 12 of the 17 samples previously analysed (Fig. 5). Single copy loci reference gene measurements were also compared with the multicopy genomic repeat *ALUJ*. For most of the samples, the apparent copy number abundances observed in the initial analysis of individual donor samples (Fig. 5) were also observed in the repeated analysis, in that *ALUJ* and *TERT* genomic copies exceeded those of *RPHH1*, which in turn were higher than those measured for *ERV3* (Fig. 7; see also the electronic supplementary material). The arithmetic mean of the log-transformed copy number measurements for the three single-copy loci was calculated (note that this is equivalent to the geometric mean using non-log-transformed copy numbers), along with the 95 % confidence interval, based on the variation between the three loci and the precision of independently replicated measurements (see “Materials and methods”). The range covered by the upper and lower limits of the expanded uncertainty occupied a central region (Fig. 7, shaded area), approximately between the higher *TERT*- and *ALUJ*-based estimates and the lower *ERV3* measurements. This suggests that the mean copy number based on multiple reference genes gave a good approximation of the possible range of cfDNA genomic copies present.

## Discussion

The potential of cfDNA in plasma as a source of minimally invasive material for molecular diagnostics is beginning to be realised. Several reports highlighting the lack of standardisation in this field [12, 13, 50] and comparison of different extraction methods have begun to elucidate the impact of different methods used on the quantification of the resulting cfDNA [18–24, 26]. In this study we have investigated cfDNA standardisation by applying different strategies to the quantification of cfDNA, including an in-house artificial spike-in control material along with targeting of endogenous (reference) genes to measure total cfDNA present in a sample.

Although it is generally accepted that most of the variation observed between different individuals will be biological, there are many technical considerations that could cause a wide range of measurement discrepancies. Our data have confirmed that one of the principal causes of technical variability comes from the different isolation methods used, where the yield and efficiencies of extracted cfDNA from the same plasma samples can differ by orders of magnitude (Figs. 1, 2). By comparing three kits designed especially for the isolation of cfDNA from plasma with the commonly used DBM extraction kit, we demonstrated that the CNA kit produced a higher yield than the other kits.

The higher yields of the CNA kit compared with the DBM kit is consistent with a recent report [19], but the DBM kit was found to be more comparable to the CNA kit by Page et al. [23], although this may be due to the different adaptations of the DBM kit for cfDNA extraction in use as the report also showed reduced cfDNA yields using the FA kit. However, by adding a plasmid spike-in control material to plasma prior to extraction, our study was able to assign a measure of extraction efficiency, further stratified by fragment size, to method performance. For the CNA kit, this suggested that nearly all cfDNA(83–100 %) was recovered. The repeatability of the extraction stage was also shown to differ between methods, with the CNA and DBM kits having a CV of less than 15 %, whereas the repeatability of NS kit was more variable (approximately 50 %).

Further technical factors whereby extraction may influence the quality of cfDNA analysis were investigated in our study. Kits developed specifically for cfDNA analysis offer the possibility of large input and/or small elution volumes. This may compound problems associated with amplification of low copy number templates by increasing the carryover of potential inhibitors into downstream analysis methods such as qPCR. The data here demonstrated that although the two kits tested (CNA and NS kits) were capable of producing highly concentrated cfDNA extracts (up to the equivalent of 250 μL plasma per microlitre of eluate and 100 μL plasma per microlitre of eluate for the CNA and NS kits, respectively), alternative qPCR detection methods were affected differently by inhibitors, with the intercalating dye method less tolerant of the highest extract concentration compared with hydrolysis probe chemistry (Fig. 3). This is in agreement with our earlier observations [51].

Cell-free (cfDNA) extractions are commonly performed on 1 mL of plasma [18, 23]. By checking the extraction efficiency with larger plasma input volumes, we have shown that total cfDNA yield increases with increased input volumes processed using the CNA kit (Fig. 4a), at least until the maximum input volume of 5 mL plasma, where a small reduction in extraction efficiency may occur (Fig. 4). It is proposed that this may be due to plasma matrix components reducing flow through the column and impacting absorption of nucleic acids [24]. However, the advantage of sample concentration with larger input volumes outweighs any slight reduction in efficiency, especially for minority detection, where larger input volumes offer the possibility of improved sensitivity of detecting the minority variant [24].

It is quoted frequently that the expected amount of cfDNA from plasma in a normal human is of the order of 1,000 genome equivalents per millilitre of blood [52]. However, not only does this value vary considerably in the literature [12, 34], but the unit used to report the value also varies (absorption units, nangrams per millilitre of plasma, etc.) and the method used to convert one unit to another (e.g. conversion of nanograms per millilitre to genome equivalents per millilitre) is based on certain assumptions, such as uniform genome representation in a sample and 3.3 pg as the weight of a human haploid genome. This conversion has relevance for quantification of targets present as single copies per haploid genome that are commonly used as reference genes for copy number variation assays. Such normalisation is required for detection of amplification or deletion in disease states, such as *ERBB2* amplification in breast cancer [10].

Likewise, the sensitivity of techniques to measure tumour-derived somatic mutations is commonly defined as a percentage of the wild-type target. Consequently, it is useful to know how many wild-type genome equivalents are present in a cfDNA sample in order to decide whether an assay is capable of measuring either the amplification of particular loci or a mutation present at 0.1 % (i.e. one copy per 1,000 genome equivalents). In these instances, the measurements of a single locus may be compromised by low technical reproducibility and biases due to its overrepresentation or underrepresentation in the cfDNA. By comparing seven different assays for commonly used reference gene loci, this study has shown the resulting estimate of total genome equivalents per millilitre of plasma can vary by over twofold in magnitude within the same normal female donor (Fig. 5). This clearly demonstrates potential problems when targeting a single genomic region for estimating the abundance of cfDNA.

The droplet dPCR validation of the qPCR reference assay measurements led us to conclude that there could be both technical and biological reasons for disparity in the copy number estimates from the different reference loci observed using qPCR measurements (Fig. 5). Although the same ranking of copy number abundances was observed for the three gene targets investigated using both approaches, the magnitude of the difference was greater using qPCR compared with droplet dPCR measurements (Fig. 6). This suggests that there may be artefacts of the standard-curve-based measurements for qPCR leading to higher copy numbers being observed for some reference genes compared with others. In this study, our standard curves were based on good-quality genomic DNA as surrogate material, for cfDNA is hard to obtain. As dPCR does not need standard curves for quantification, this approach removes this potential source of bias from the data.

The similarity in ranking between qPCR and droplet dPCR measurements suggests that different loci are present at different proportions in the cell-free fraction from those present in cellular genomic DNA. Of note is that loci that are more telomeric in location (*TERT* and *GAPDH*) are more abundant than those with a more centromeric position (*ERV3* and *RPPH1*), with *NAGK*, which is centrally located in the short arm of chromosome 2, ranked in-between. *ALUJ* is ranked level with *TERT*; this is consistent with the predominant telomeric location of these repetitive sequences [53]. Two recent studies using next-generation sequencing have demonstrated that the entire fetal genome can be isolated from the cfDNA in maternal plasma [54, 55]. This demonstrates that although there may be little or no bias in the presence or absence of certain genomic sequences, a single genomic locus or element cannot necessarily meet the requirements to standardise quantitative measurement of the tumour- or fetal-derived cfDNA fraction to the total cfDNA pool.

To generate a more robust method for standardised determination of the genomic content of cfDNA, this study applied the widely accepted best practice principle for gene expression analysis of multiple reference gene normalisation [46, 56] (Fig. 7). The GeNorm factor, or multigene average value, is the geometric mean of the copy number quantities for each of the constituent reference gene assays [46]. This is mathematically equivalent to the arithmetic mean of log-transformed quantities (as presented here). By applying the principle of GeNorm normalisation to cfDNA analysis, we calculated an average measurement that avoids a systematic overestimation or underestimation of total cfDNA load. We also outlined how the confidence interval of cfDNA load may be calculated from the contributions from reference gene variability and assay precision (based on independent qPCR measurements) and demonstrated that this approach is consistent with any of the single-gene estimates of cfDNA quantity (Fig. 7). Hindson et al. [44] also applied the principle of multiple reference gene averaging for quantification of total cfDNA for non-invasive prenatal diagnosis using four genetic loci using droplet dPCR. In this case, the contribution of each target to the overall average was weighted according to assay precision.

Calculating the confidence interval or ‘measurement uncertainty’ associated with total cfDNA load may help to characterise the sample prior to multiple downstream analyses (e.g. screening for several different biomarkers) and guide clinical boundaries relating to the concentration, and measurable differences, of a specific biomarker. For example, the mean and the confidence interval for total cfDNA load for donor 1 (Fig. 7) were 1,400 copies per millilitre of plasma and 500–3,800 copies per millilitre of plasma, respectively. If a mutant biomarker is present at 14 copies per millilitre, the confidence interval associated with the total cfDNA load would predict an abundance of the biomarker of between 0.4 and 2.7 % (mean 1 %). Figure 7 shows our estimate of the confidence interval may be conservative, as we included reference genes with the largest difference in copy number ranking (*TERT* and *ERV3*). Using three reference genes also means that the result has two degrees of freedom, and the associated confidence intervals are based on a Student’s *t* value of 4.3. Inclusion of additional genomic loci in the reference gene panel could improve the confidence of the estimate of cfDNA load, and this may be necessary if more precise measurements are required (e.g. when measuring smaller fold changes).

We therefore recommend that, in the same way as reference messenger RNAs are selected on the basis of their stability for gene of interest normalisation [57, 58], a panel of genomic reference loci be screened in samples from the intended clinical cohort in order to ensure that a representative cross-section of the genome is measured in order to assign cfDNA load.

## Conclusions

The potential of cfDNA as an analyte for tumour and prenatal diagnostics as well as a prognostic indicator for transplant rejection [59] and sepsis [60] is becoming increasingly apparent. A number of different techniques are being applied to circulating cfDNA analysis, from targeted approaches such as dPCR [61, 62] and deep sequencing of mutation hotspots [63], to whole exome and whole genome sequencing [64, 65]. Accurate quantification of total cfDNA will aid future clinical implementation of such approaches through quality assurance of technical performance (e.g. ensuring input quantity is sufficient to achieve the required sequencing depth). For non-invasive prenatal diagnosis of fetal aneuploidies, it is also important to characterise the fraction of total cfDNA which originates from the fetus [66].

In this study, we have investigated the efficiency of methods for extraction of cfDNA from plasma and have demonstrated how a spike-in containing fragment sizes relevant to cfDNA can be used to assess recovery of differently sized DNA. We have applied a strategy developed for standardisation of messenger RNA to cfDNA load measurements using multiple reference genes to minimise biases due to assay or genome location. This report is timely in raising awareness of the need, and establishing a benchmark, for standardisation of the extraction and quantification of cfDNA in these exciting fields of molecular diagnostics.

## Electronic supplementary material

Below is the link to the electronic supplementary material.

ESM 1 (PDF 1.66 MB)
