# Supplementary material for: Towards standardisation of cell-free DNA measurement in plasma: controls for extraction efficiency, fragment size bias and quantification
Source: Anal Bioanal Chem. 2014 May 24;406(26):6499–512. doi: 10.1007/s00216-014-7835-3 (PMC4182654; doi:10.1007/s00216-014-7835-3)
Supplement: Supplementary file 1 — (PDF 1.66 MB) [file 216_2014_7835_MOESM1_ESM.pdf]

Analytical and Bioanalytical Chemistry

Electronic Supplementary Material

Towards standardisation of cell-free DNA measurement in plasma: controls for extraction efficiency, fragment size bias and quantification

Alison S. Devonshire, Alexandra S. Whale, Alice Gutteridge, Gerwyn Jones, Simon Cowen, Carole A. Foy, Jim F. Huggett

Fig. S1. Preparation of fragmented *ADH* plasmid

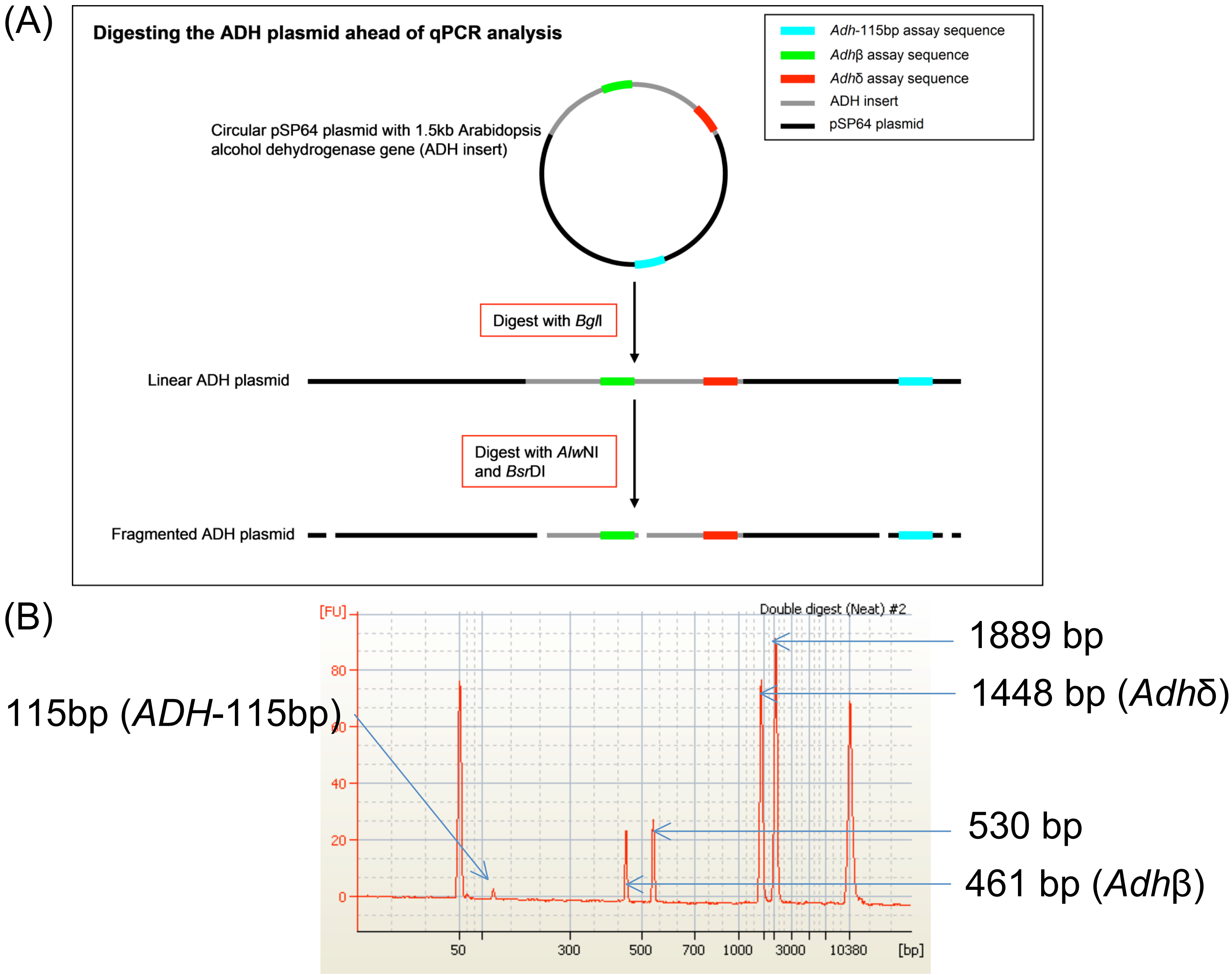

Fig. S2. Inhibition testing of cfDNA extracts from CNA, NS and FA kits

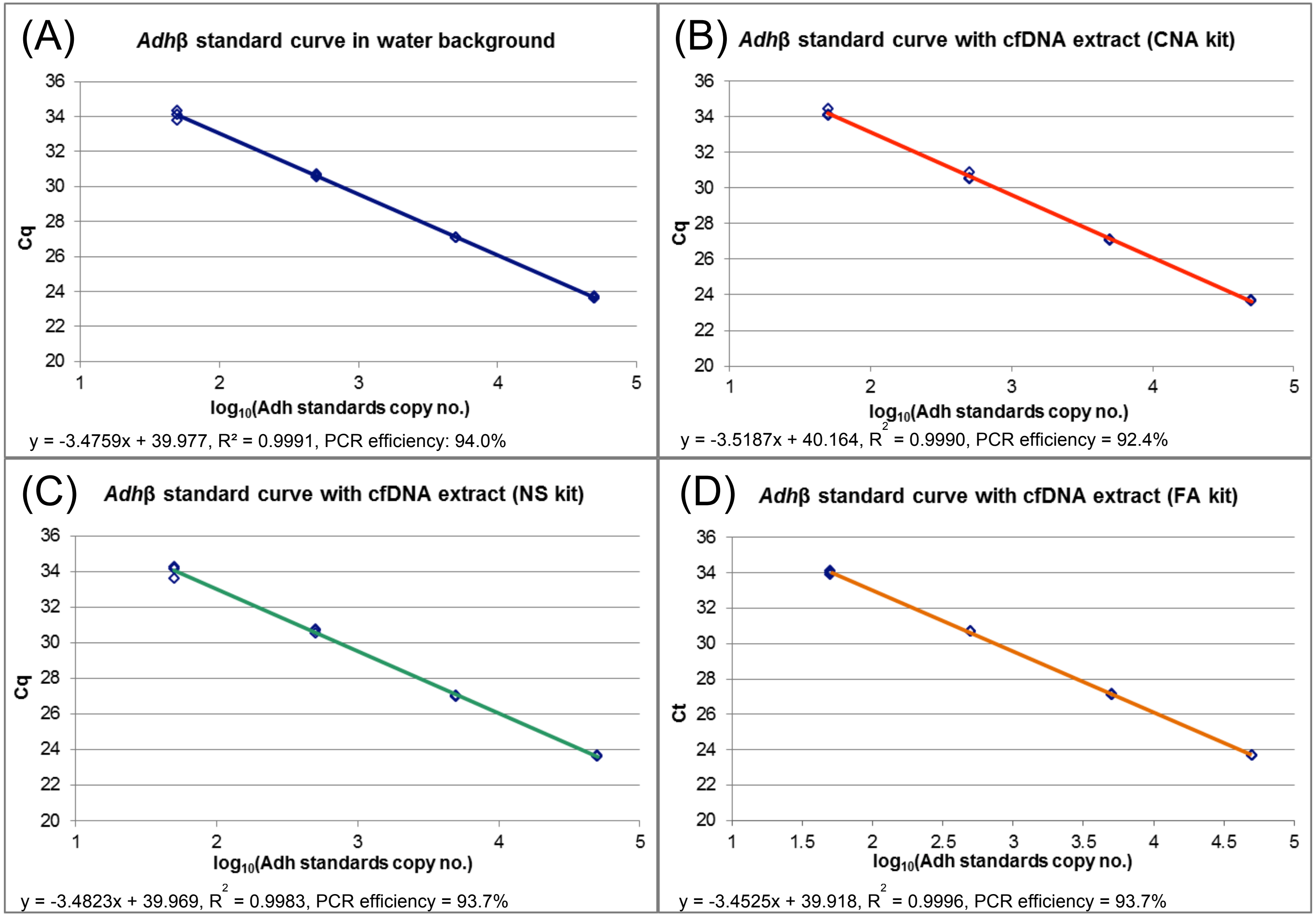

Fig. S3. cfDNA genomic copy numbers measured in extracts from 17 donors

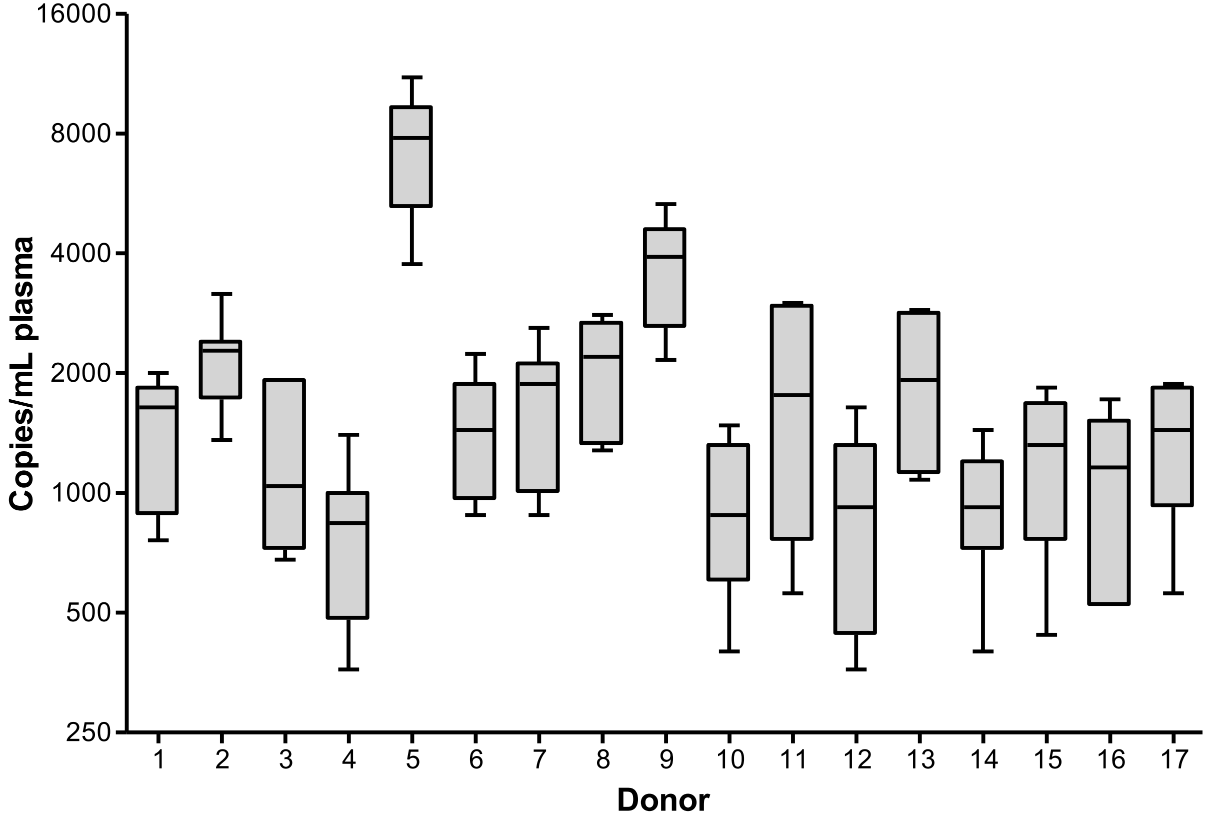

Fig. S4. Optimisation of droplet dPCR experiments

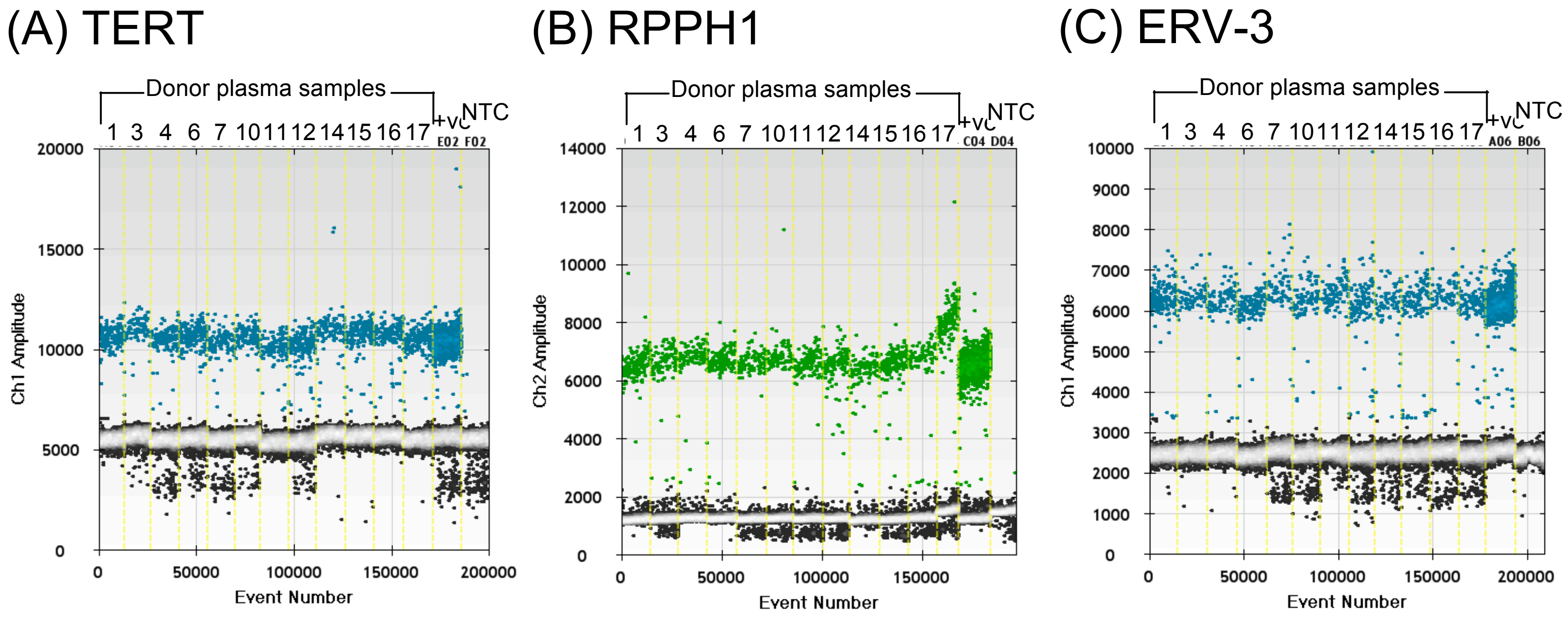

Fig. S5. Comparison of reference gene copy numbers measured by qPCR and droplet dPCR in cfDNA extracts from 12 plasma samples

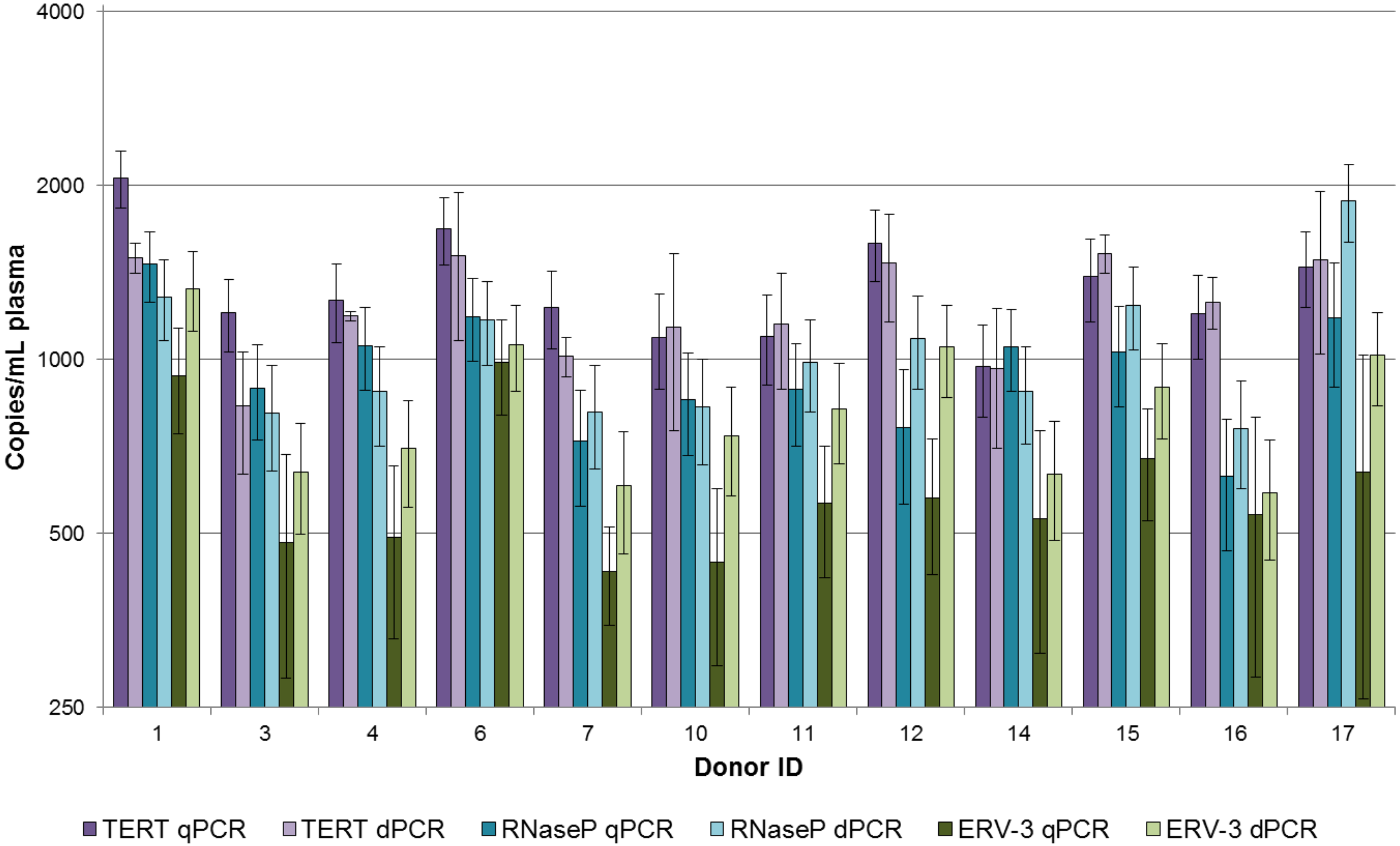

Table S1. Plasma sample information and composition of plasma pools

Donor information			Plasma pool		
**Number**	**Age**	**Race**	**A (i)**	**A (ii)**	**B**
1	59	C	✓	✓	
2	53	B	✓	✓	
3	54	B	✓	✓	
4	55	H	✓	✓	
5	52	B	✓	✓	
6	59	B			✓
7	50	B			✓
8	53	B			✓
9	51	C			✓
10	50	H			✓
11	50	B			✓
12	53	B			✓
13	54	B			✓
14	52	B			✓
15	53	B			✓
16	54	B			✓
17	50	B			✓

Details of each donor are given with the relevant plasma pools. For the race key, C: Caucasian, B: Black and H: Hispanic.

Table S2. qPCR assay information

Gene Symbol/ Assay name	Genome loci/ADH fragment size	Location	Primer/probe sequence (5'-3')	[Primer/ probe] (μM)	Hydrolysis probe fluorophore /quencher	Commercial mastermix†	Amplicon length (bp)	Average efficiency ± SD*	R^2*	Intra-assay repeatability SD Cq*	LOD (GE or plasmid copies as appropriate)	SD Cq (LOD)
TERT	5p15.33	NC_000005.9 (1253282.. 1295178, complement)	F: CCTCACATAAATGCTACCAAACGA	0.9	FAM/BHQ1	Universal	79	98.17% ± 2.36% (n=7)	0.994 (n=7)	0.13	5	0.48
			R: TTCCAAGAAGGAGGCCATAGTC	0.9								
			P: AAGAAATGAACAGACCCATCCCCCAGG	0.25								
ALUJ	N/A	N/A	F: CAACATAGTGAAACCCCGTCTCT	0.6	N/A	Power SYBR	N/A	92.55% ± 1.81% (n=5)	0.998 (n=5)	0.18	≤ 0.2	0.25
			R: GCCTCAGCCTCCCGAGTAG	0.6								
RPPH1	14q11.2	NC_000014.8 (20811230.. 20811570, complement)	F: GCGGAGGGAAGCTCATCAG	0.9	VIC/MGB	Universal	64	96.07% ± 4.36% (n=5)	0.995 (n=5)	0.10	5	0.24
			R: GGACATGGGAGTGGAGTGACA	0.9								
			P: CACGAGCTGAGTGCG	0.2								
GAPDH	12p13	NC_000012.11 (6643585.. 6647537)	F: AGGTTTACATGTTCCAATATGATTCCA	0.45	FAM-BHQ1	Universal	94	100.51% ± 6.94% (n=2)	0.996 (n=2)	0.10	5	0.36
			R: ATGGGATTTCCATTGATGACAAG	0.45								
			P: CCGTTCTCAGCCTTGACGGTGC	0.225								
NAGK	2p13.3	NC_000002.11 (71295408.. 71305998)	F: TGGGCAGACACATCGTAGCA	0.2	FAM-BHQ1	Universal	66	101.33% ± 2.99% (n=2)	0.989 (n=2)	0.17	5	0.81
			R: CACCTTCACTCCCACCTCAAC	0.2								
			P: TGTTGCCCGAGATTGACCCGGT	0.1								
ERV-3	7q11.2	NC_000007.13 (64450733.. 64467124, complement)	F: CATGGGAAGCAAGGGAACTAATG	0.2	FAM-BHQ1	Universal	135	99.34% ± 4.85% (n=5)	0.994 (n=5)	0.16	5	0.76
			R: CCCAGCGAGCAATACAGAATTT	0.2								
			P: TCTTCCCTCGAACCTGCACCATCAA	0.15								
Valid Prime	Proprietary	Proprietary	Primer mix	0.4	FAM-BHQ1	Universal	Proprietary	91.57% ± 0.82% (n=2)	0.992 (n=2)	0.16	5	0.61
			Probe solution	0.2								
*Adh*β	461 bp	M12196	F: TTGAGAGTGTTGGAGAAGGAGTGA	0.9	FAM/MGB	Universal	461	95.65% ± 5.17% (n=4)	0.998 (n=4)	0.03	50	0.21
			R: CGGTAAAGATCGGCAACACA	0.9								
			P: TCTTCAGCCAGGAGATC	0.2								
*Adh*δ	1448 bp	M12196	F: TGAACCCGAAAGACCATGACA	0.9	FAM/MGB	Universal	1448	97.11% ± 2.62% (n=3)	0.998 (n=3)	0.12	50	0.26
			R: CCCACCATCCGTCATCTCA	0.9								
			P: CCAATTCAACAGGTGATC	0.2								
ADH- 115bp	115 bp	M12196	F: GGGCCGAGCGCAGAA	0.9	FAM/BHQ1	Gene Expression	115	93.42% ± 2.09% (n=5)	0.996 (n=5)	0.13	5	0.43
			R: ACTCTAGCTTCCCGGCAACA	0.9								
			P: TGGTCCTGCAACTTTATCCGCCTCC	0.25								

*For assay optimisation, the number of replicate experiments (n) is given which were used to determine mean PCR efficiencies and R^2 values.

**SD values given for 125 GE/reaction (human genomic targets), 1 GE/reaction (ALUJ) or 500 copies/reaction (*ADH* assays).

†PCR mastermixes (all Life Technologies): TaqMan® Universal PCR Master Mix (with AmpErase® UNG), Power SYBR® Green PCR Master Mix, TaqMan® Gene Expression Master Mix.

Table S3. (A) MIQE checklist

ITEM TO CHECK	IMPORTANCE	CHECKLIST
**EXPERIMENTAL DESIGN**		
Definition of experimental and control groups	**E**	Materials & Methods and Table S1
Number within each group	**E**	Materials & Methods and Table S1
Assay carried out by core lab or investigator's lab?	D	Investigator's lab
Acknowledgement of authors' contributions	D	Author information
**SAMPLE**		
Description	**E**	Materials & Methods and Table S1
Volume/mass of sample processed	D	Materials & Methods and Table S1
Microdissection or macrodissection	**E**	N/A
Processing procedure	**E**	Materials & Methods
If frozen - how and how quickly?	**E**	Materials & Methods
If fixed - with what, how quickly?	**E**	N/A
Sample storage conditions and duration (especially for FFPE samples)	**E**	Experiments performed within 6 months of sample receipt
**NUCLEIC ACID EXTRACTION**		
Procedure and/or instrumentation	**E**	Materials & Methods
Name of kit and details of any modifications	**E**	Materials & Methods
Source of additional reagents used	D	N/A
Details of DNase or RNAse treatment	**E**	No RNase/DNase treatment required
Contamination assessment (DNA or RNA)	**E**	N/A
Nucleic acid quantification	**E**	qPCR/dPCR
Instrument and method	**E**	Materials & Methods
Purity (A260/A280)	D	N/A (concentration too low)
Yield	D	Results
RNA integrity method/instrument	**E**	N/A
RIN/RQI or Cq of 3' and 5' transcripts	**E**	N/A
Electrophoresis traces	D	N/A
Inhibition testing (Cq dilutions, spike or other)	**E**	ADH spike-in. See Materials & Methods, Figure 3 and Figure S2
**REVERSE TRANSCRIPTION**		
Complete reaction conditions	**E**	N/A
Amount of RNA and reaction volume	**E**	N/A
Priming oligonucleotide (if using GSP) and concentration	**E**	N/A
Reverse transcriptase and concentration	**E**	N/A
Temperature and time	**E**	N/A
Manufacturer of reagents and catalogue numbers	D	N/A
Cqs with and without RT	D*	N/A
Storage conditions of cDNA	D	N/A

Table S3. (A) MIQE checklist (cont.)

qPCR TARGET INFORMATION		
If multiplex, efficiency and LOD of each assay.	**E**	N/A
Sequence accession number	**E**	Table S2
Location of amplicon	D	Not included
Amplicon length	**E**	Table S2
In silico specificity screen (BLAST, etc)	**E**	NCBI PrimerBlast
Pseudogenes, retropseudogenes or other homologs?	D	N/D
Sequence alignment	D	N/D
Secondary structure analysis of amplicon	D	N/D
Location of each primer by exon or intron (if applicable)	**E**	N/A (DNA)
What splice variants are targeted?	**E**	N/A (DNA)
**qPCR OLIGONUCLEOTIDES**		
Primer sequences	**E**	Table S2
RTPrimerDB Identification Number	D	N/A
Probe sequences	D**	Table S2
Location and identity of any modifications	**E**	Table S2
Manufacturer of oligonucleotides	D	Sigma Aldrich (excl. ValidPrime (TATAA Biocentre) and MGB probes (ABI))
Purification method	D	HPLC
**qPCR PROTOCOL**		
Complete reaction conditions	**E**	Materials & Methods, Table S2
Reaction volume and amount of cDNA/DNA	**E**	Materials & Methods
Primer, (probe), Mg++ and dNTP concentrations	**E**	Table S2
Polymerase identity and concentration	**E**	*Taq*
Buffer/kit identity and manufacturer	**E**	Table S2
Exact chemical constitution of the buffer	D	Proprietary
Additives (SYBR Green I, DMSO, etc.)	**E**	No additives
Manufacturer of plates/tubes and catalog number	D	96-well: 4306737 384-well: 4309849
Complete thermocycling parameters	**E**	Materials & Methods
Reaction setup (manual/robotic)	D	Manual
Manufacturer of qPCR instrument	**E**	Materials & Methods
**qPCR VALIDATION**		
Evidence of optimisation (from gradients)	D	Table S2
Specificity (gel, sequence, melt, or digest)	**E**	Melt curve analysis (SYBR Green)
For SYBR Green I, Cq of the NTC	**E**	Table S4
Standard curves with slope and y-intercept	**E**	Table S2
PCR efficiency calculated from slope	**E**	Table S2
Confidence interval for PCR efficiency or standard error	D	N/D
r2 of standard curve	**E**	Table S2
Linear dynamic range	**E**	Table S2
Cq variation at lower limit	**E**	Table S2
Confidence intervals throughout range	D	N/D
Evidence for limit of detection	**E**	Standard curve
If multiplex, efficiency and LOD of each assay.	**E**	N/A

Table S3. (A): MIQE checklist (cont.)

DATA ANALYSIS		
qPCR analysis program (source, version)	**E**	Materials & Methods
Cq method determination	**E**	Materials & Methods
Outlier identification and disposition	**E**	Grubb's test
Results of NTCs	**E**	Table S4
Justification of number and choice of reference genes	**E**	Results
Description of normalisation method	**E**	Results
Number and concordance of biological replicates	D	N/A (no biological replicate plasma samples)
Number and stage (RT or qPCR) of technical replicates	**E**	Materials & Methods
Repeatability (intra-assay variation)	E	Table S2
Reproducibility (inter-assay variation, %CV)	D	Results
Power analysis	D	N/A
Statistical methods for result significance	**E**	Materials & Methods
Software (source, version)	E	Materials & Methods
Cq or raw data submission using RDML	**D**	N/D

MIQE checklist for authors, reviewers and editors. All essential information (E) must be submitted with the manuscript. Desirable information (D) should be submitted if available. If using primers obtained from RTPrimerDB, information on qPCR target, oligonucleotides, protocols and validation is available from that source.

*Assessing the absence of DNA using a no RT assay is essential when first extracting RNA. Once the sample has been validated as RDNA-free, inclusion of a no-RT control is desirable, but no longer essential.
**Disclosure of the probe sequence is highly desirable and strongly encouraged. However, since not all commercial pre-designed assay vendors provide this information, it cannot be an essential requirement. Use of such assays is advised against.

Table S3. (B) dMIQE checklist

ITEM TO CHECK	IMPORTANCE	CHECKLIST
**dPCR PROTOCOL**		
Complete reaction conditions	E	Materials & Methods
Reaction volume and amount of cDNA/DNA	E	Materials & Methods
Primer, (probe), Mg++ and dNTP concentrations	E	Materials & Methods
Polymerase identity and concentration	E	Materials & Methods
Buffer/kit identity and manufacturer	E	Materials & Methods
Exact chemical constitution of the buffer	D	Proprietary
Additives (SYBR Green I, DMSO, etc.)	E	No additives
Plates/tubes catalogue number and manufacturer	D	Materials & Methods
Complete thermocycling parameters	E	Materials & Methods
Reaction setup (manual/robotic)	D	Manual
Gravimetric or volumetric dilutions (manual/robotic)	D	N/A
Total PCR volume prepared	D	Materials & Methods
Partition number	E	Table S5
Individual partition volume	E	0.91 nL according to manufacturer
Total volume of the partitions measured (effective reaction size)	E	Table S5
Partition volume variance/SD	D	Unknown
Comprehensive details and appropriate use of controls	E	Materials & Methods
Manufacturer of dPCR instrument	E	Materials & Methods
**dPCR VALIDATION**		
Optimisation data for the assay	D	Table S2
Specificity (when measuring rare mutations, pathogen sequences etc)	E	N/A
Limit of detection of calibration control	D	N/A
If multiplexing, comparison with singleplex assays	E	N/A
**DATA ANALYSIS**		
Mean copies per partition (λ or equivalent)	E	Table S5
dPCR analysis prgram (source, version)	E	Materials & Methods
Outlier identification and disposition	E	N/A
Results of NTCs	E	Table S5
Examples of positive(s) and negative experimental results as supplemental data	E	Supplementary Figure 4
Where appropriate, justification of number and choice of reference genes	E	Results
Where appropriate, description of normalization method	E	Results
Number and concordance of biological replicates	D	N/A (no biological replicate plasma samples)
Number and stage (RT or qPCR) of technical replicates	E	Figure legends
Repeatability (intra-assay variation)	E	Table S2
Reproducibility (inter-assay/user/lab etc variation)	D	N/D
Experimental variance or CI [d]	E	Table S5
Statistical methods for analysis	E	Materials & Methods
Data submission using RDML (Real-time PCR Data Markup Language)	D	Table S5

dMIQE checklist for authors, reviewers and editors. All essential information (E) must be submitted with the manuscript. Desirable information (D) should be submitted if possible.

Due to the overlap with the MIQE guidelines (Table S3A), items to check under Experimental Design, Sample, Nucleic Acid Extraction, Target Information and Oligonucleotides have been ommited from this table but are part of the dMIQE guidlines.

[d] When single dPCR experiments are performed, the variation due to counting error alone should be calculated from the Binomial (or suitable equivalent) distribution.

Table S4. Cq values of NTCs

Figure	Assay	Cq value	Internal reference
Figure 1	ALUJ	29.81	Exp015
Figure 1	ALUJ	29.75	Exp015
Figure 1	ALUJ	29.63	Exp015
Figure 1	TERT	Undetermined	Exp015
Figure 1	TERT	Undetermined	Exp015
Figure 1	TERT	Undetermined	Exp015
Figure 2	Adhβ (Taqman)	Undetermined	Exp010c
Figure 2	Adhβ (Taqman)	Undetermined	Exp010c
Figure 2	Adhδ (Taqman)	Undetermined	Exp010c
Figure 2	Adhδ (Taqman)	Undetermined	Exp010c
Figure 2	ADH plasmid 115 bp	37.02	Exp010c repeat
Figure 2	ADH plasmid 115 bp	Undetermined	Exp010c repeat
Figure 2	ADH plasmid 115 bp	36.99	Exp010c repeat
Figure 3	Adhβ (Taqman)	Undetermined	Exp12
Figure 3	Adhβ (Taqman)	Undetermined	Exp12
Figure 3	Adhβ (Taqman)	Undetermined	Exp12
Figure 3	Adhβ (SYBR)	Undetermined	Exp12
Figure 3	Adhβ (SYBR)	37.45	Exp12
Figure 3	Adhβ (SYBR)	Undetermined	Exp12
Figure 4	TERT	Undetermined	Exp29a
Figure 4	TERT	Undetermined	Exp29a
Figure 4	TERT	Undetermined	Exp29a
Figure 5	TERT	Undetermined	Exp22
Figure 5	TERT	Undetermined	Exp22
Figure 5	TERT	Undetermined	Exp22
Figure 5	ALUJ	27.73	Exp22
Figure 5	ALUJ	28.15	Exp22
Figure 5	ALUJ	27.86	Exp22
Figure 5	RPPH1	Undetermined	Exp22
Figure 5	RPPH1	Undetermined	Exp22
Figure 5	RPPH1	Undetermined	Exp22
Figure 5	GAPDH	Undetermined	Exp22
Figure 5	GAPDH	Undetermined	Exp22
Figure 5	GAPDH	Undetermined	Exp22
Figure 5	NAGK	Undetermined	Exp22
Figure 5	NAGK	Undetermined	Exp22
Figure 5	NAGK	Undetermined	Exp22
Figure 5	ERV-3	Undetermined	Exp22
Figure 5	ERV-3	Undetermined	Exp22
Figure 5	ERV-3	Undetermined	Exp22
Figure 5	Valid Prime	Undetermined	Exp22
Figure 5	Valid Prime	Undetermined	Exp22
Figure 5	Valid Prime	Undetermined	Exp22

Table S4. Cq values of NTCs (cont.)

Figure	Assay	Cq value	Internal reference
Figures 6&7	TERT	Undetermined	Exp025a
Figures 6&7	TERT	Undetermined	Exp025b
Figures 6&7	TERT	Undetermined	Exp025c
Figures 6&7	ALU J	29.96	Exp025a
Figures 6&7	ALU J	29.87	Exp025b
Figures 6&7	ALU J	30.18	Exp025c
Figures 6&7	RPPH1	Undetermined	Exp025a
Figures 6&7	RPPH1	Undetermined	Exp025b
Figures 6&7	RPPH1	Undetermined	Exp025c
Figures 6&7	ERV-3	Undetermined	Exp025a
Figures 6&7	ERV-3	Undetermined	Exp025b
Figures 6&7	ERV-3	Undetermined	Exp025c

Table S5. Droplet dPCR data

Plasma sample	Assay	Positive partitions	Number of partitions	Total volume of partitions (μL)	λ	Quant. (c/μL)	95% CI-L (c/μL)	95% CI-H (c/μL)
1	TERT	161	13563	12.34	0.01	74.99	63.41	86.58
3	TERT	88	13326	12.13	0.01	41.60	32.92	50.30
4	TERT	139	14749	13.42	0.01	59.46	49.58	69.35
6	TERT	175	14629	13.31	0.01	75.57	64.38	86.78
7	TERT	110	13694	12.46	0.01	50.64	41.19	60.12
10	TERT	119	13186	12.00	0.01	56.93	46.71	67.16
11	TERT	131	14373	13.08	0.01	57.50	47.66	67.35
12	TERT	166	14253	12.97	0.01	73.56	62.38	84.76
14	TERT	114	14918	13.58	0.01	48.17	39.33	57.02
15	TERT	174	14404	13.11	0.01	76.32	64.99	87.67
16	TERT	151	15174	13.81	0.01	62.80	52.79	72.83
17	TERT	180	15306	13.93	0.01	74.28	63.44	85.15
NTC	TERT	3	13907	12.66	0.00	1.35	-0.18	2.89
1	RPPH1	152	14950	13.60	0.01	64.17	53.98	74.38
3	RPPH1	89	13875	12.63	0.01	40.41	32.02	48.81
4	RPPH1	99	14177	12.90	0.01	44.00	35.34	52.68
6	RPPH1	139	15001	13.65	0.01	58.46	48.75	68.18
7	RPPH1	94	14605	13.29	0.01	40.55	32.35	48.75
10	RPPH1	89	13536	12.32	0.01	41.42	32.82	50.04
11	RPPH1	117	14901	13.56	0.01	49.50	40.54	58.48
12	RPPH1	114	13220	12.03	0.01	54.38	44.41	64.38
14	RPPH1	104	14847	13.51	0.01	44.14	35.66	52.63
15	RPPH1	144	14637	13.32	0.01	62.08	51.95	72.23
16	RPPH1	85	14126	12.85	0.01	37.90	29.85	45.96
17	RPPH1	163	10956	9.97	0.01	94.13	79.69	108.59
NTC	RPPH1	1	12470	11.35	0.00	0.50	-0.48	1.49
1	ERV-3	157	14926	13.58	0.01	66.40	56.02	76.80
3	ERV-3	81	15998	14.56	0.01	31.87	24.94	38.82
4	ERV-3	88	15781	14.36	0.01	35.11	27.78	42.46
6	ERV-3	132	15698	14.29	0.01	53.03	43.99	62.08
7	ERV-3	67	13938	12.68	0.00	30.26	23.02	37.51
10	ERV-3	85	14515	13.21	0.01	36.88	29.04	44.73
11	ERV-3	99	15172	13.81	0.01	41.11	33.02	49.21
12	ERV-3	116	13917	12.66	0.01	52.56	43.00	62.13
14	ERV-3	71	14091	12.82	0.01	31.72	24.35	39.10
15	ERV-3	108	15187	13.82	0.01	44.81	36.37	53.27
16	ERV-3	70	14989	13.64	0.00	29.39	22.51	36.28
17	ERV-3	114	14120	12.85	0.01	50.90	41.57	60.26
NTC	ERV-3	0	15377	13.99	0.00	0.00	0.00	0.00

Table S6. Comparison of kits for cfDNA extraction: % Coefficients of variation

		Kit		
**Target**	**Assay**	**CNA**	**NS**	**DBM**
Endogenous	*TERT*	12%	47%	10%
Endogenous	*ALUJ*	12%	46%	23%
ADH spike-in	1448 bp	12%	58%	2%
ADH spike-in	461 bp	4%	55%	11%
ADH spike-in	115 bp	8%	40%	5%
Mean	All	10%	49%	10%

Table S7. Correlation analysis of reference gene assays

Copy number values for each donor QX1-QX17 (Figure 5) were log10 transformed. Results of Pearson pairwise correlation analysis (r and p values) are displayed below).

R-values

Assay	**ALUJ**	**ERV3**	**GAPDH**	**NAGK**	**RPPH1**	**TERT**	**VP**
ALUJ		0.900	0.946	0.910	0.896	0.952	0.861
ERV3	0.900		0.950	0.919	0.890	0.922	0.910
GAPDH	0.946	0.950		0.929	0.904	0.938	0.907
NAGK	0.910	0.919	0.929		0.888	0.910	0.931
RPPH1	0.896	0.890	0.904	0.888		0.966	0.860
TERT	0.952	0.922	0.938	0.910	0.966		0.894
VP	0.861	0.910	0.907	0.931	0.860	0.894	

p-values

	**ALUJ**	**ERV3**	**GAPDH**	**NAGK**	**RPPH1**	**TERT**	**VP**
**ALUJ**		8.52467E-07	9.95E-09	3.95E-07	1.18241E-06	4.28E-09	9.21302E-06
**ERV3**	8.52467E-07		5.96E-09	1.92E-07	1.73279E-06	1.45E-07	4.10E-07
**GAPDH**	9.95E-09	5.96E-09		7.08E-08	6.49888E-07	2.64E-08	5.08022E-07
**NAGK**	3.95E-07	1.92E-07	7.08E-08		2.00408E-06	4.04E-07	5.99E-08
**RPPH1**	1.18241E-06	1.73279E-06	6.49888E-07	2.00408E-06		3.44E-10	9.38501E-06
**TERT**	4.28E-09	1.45E-07	2.64E-08	4.04E-07	3.44E-10		1.33382E-06
**VP**	9.21302E-06	4.10E-07	5.08022E-07	5.99E-08	9.38501E-06	1.33382E-06	

Table S8. GeNorm analysis of 7 reference genes in 17 donor samples

Input: Copy numbers

**Gene Name**	**M-Value (smallest value, most stable)**
ERV-3	0.390
RPHH1	0.368
VP	0.338
NAGK	0.316
TERT	0.295
GAPDH	0.293
ALU J	0.293

Supplementary Methods (Statistical analysis)

Ranking of copy number measurements according to reference gene

A log transform of the data expressed as cfDNA genomic copies/mL plasma was used to produce a residual distribution closer to Normal. Classical linear models were used to estimate coefficients and significance of terms. The significance of estimated differences between the reference genes was assessed using Tukey's Honest Significant Differences (HSD) test, and the results used to construct a rank order.

Figure 5 data (Internal reference: Experiment 22)

The Tukey HSD plot is given in Appendix A (Figure 1). This shows confidence intervals based on multiple testing for the differences between the different factor levels (in this case the 7 reference genes in the set – a total of 21 comparisons). Confidence intervals which include zero are indicative of no significant difference between a pair of Markers, and by examining the plot as a whole, it is possible to derive a rank order in terms of the observed copy number (high to low):

1. TERT, AluJ
3. NAGK, GAPDH
5. RPPH1 (RNaseP)
6. ERV3, VP (ValidPrime)

This order is consistent with the calculated differences, which are also given in Appendix B. The difference between RPPH1 and NAGK, GAPDH is very borderline ($0.04 < p < 0.05$).

Figure 6-7 qPCR data (Internal reference: Experiment 25)

This was carried out for 4 reference genes over 3 runs in a balanced experiment. One data point was found to be missing, but this is not expected to affect the estimates appreciably.

All but one of the comparisons showed differences which were strongly significantly different from zero (

Supplementary **Methods Appendix A: Figures (continued)**

Figure 2). This resulted in a rank order as follows:

1. TERT, AluJ
3. RPPH1
4. ERV3

Figure 6 ddPCR data (Internal reference: Experiment 32)

Figure 3 shows the differences, which produces the following rank order:

1 TERT

2 RPPH1

3 ERV3

The difference between TERT and RPPH1 is very borderline ($p = 0.050$).

Supplementary Methods Appendix A: Figures

Figure 1: Tukey's HSD plot for Experiment 22, showing differences between the reference genes (95% family-wise confidence level). There are 21 pairwise comparisons between the 7 in the set.

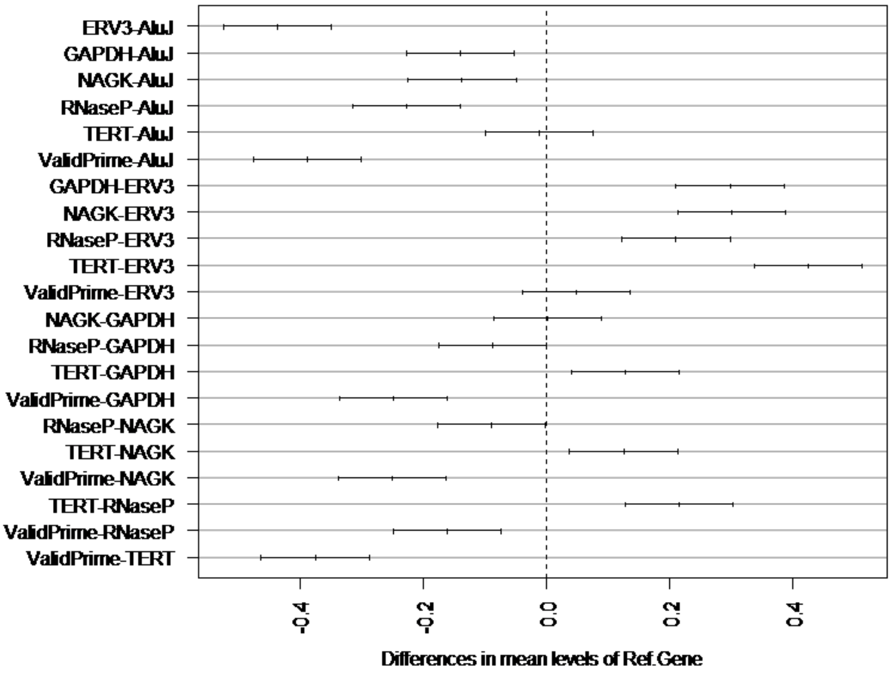

Supplementary Methods Appendix A: Figures (continued)

Figure 2: As for Figure 1, for Experiment 25.

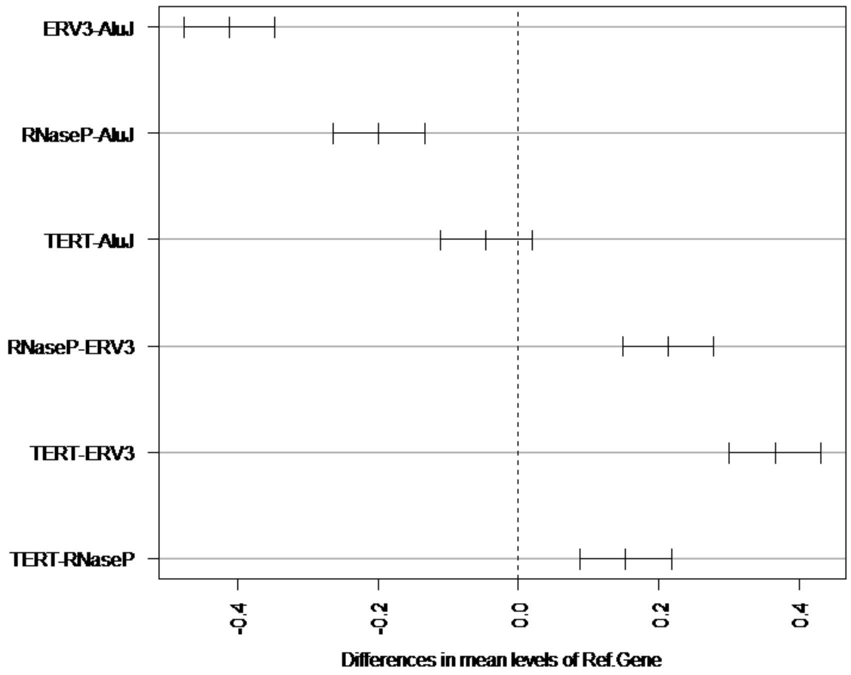

Figure 3: As for Figure 1, for Experiment 32.

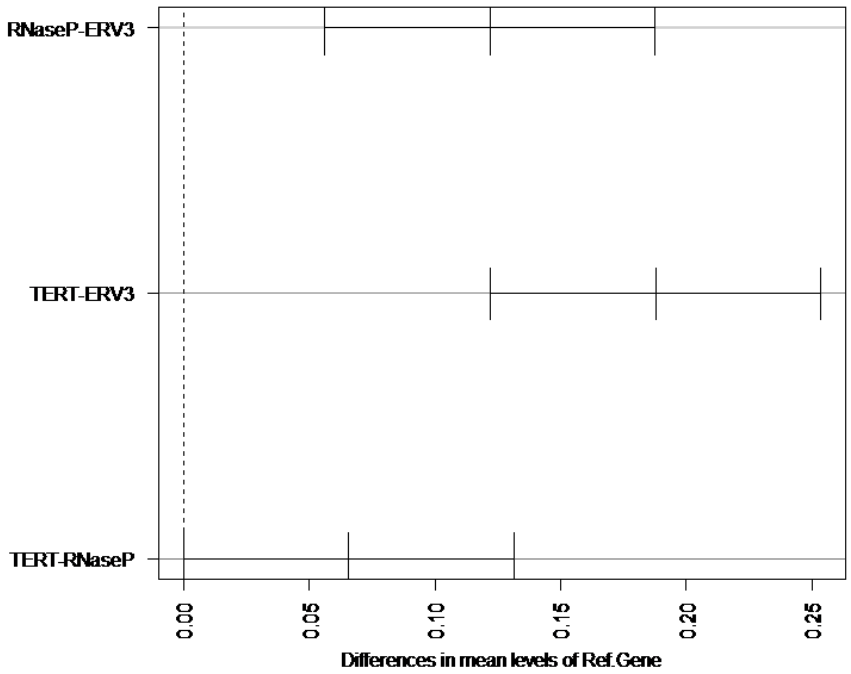

Supplementary Methods Appendix B – Model output

Experiment 22

```
Summary:
Call:
lm(formula = log10(Copies) ~ Ref.Gene + Donor - 1, data = exp22)

Residuals:
      Min        1Q    Median        3Q       Max
-0.178250 -0.049013  0.002288  0.051177  0.172693

Coefficients:
                   Estimate Std. Error t value Pr(>|t|)
Ref.GeneAluJ        3.30972    0.03723  88.904  < 2e-16 ***
Ref.GeneERV3        2.87288    0.03723  77.170  < 2e-16 ***
Ref.GeneGAPDH       3.17036    0.03723  85.160  < 2e-16 ***
Ref.GeneNAGK        3.17245    0.03723  85.217  < 2e-16 ***
Ref.GeneRPPH1      3.08283    0.03723  82.809  < 2e-16 ***
Ref.GeneTERT        3.29724    0.03723  88.569  < 2e-16 ***
Ref.GeneValidPrime  2.92154    0.03723  78.477  < 2e-16 ***
DonorQX10          -0.19880    0.04526  -4.392 2.89e-05 ***
DonorQX11           0.05433    0.04526   1.200  0.23296
DonorQX12          -0.19159    0.04526  -4.233 5.28e-05 ***
DonorQX13           0.14500    0.04526   3.203  0.00184 **
DonorQX14          -0.17963    0.04526  -3.968  0.00014 ***
DonorQX15          -0.08531    0.04526  -1.885  0.06250 .
DonorQX16          -0.14358    0.04526  -3.172  0.00203 **
DonorQX17          -0.03401    0.04526  -0.751  0.45431
DonorQX2            0.21438    0.04526   4.736 7.52e-06 ***
DonorQX3           -0.07879    0.04526  -1.741  0.08493 .
DonorQX4           -0.25282    0.04526  -5.586 2.17e-07 ***
DonorQX5            0.73043    0.04526  16.137  < 2e-16 ***
DonorQX6            0.03416    0.04526   0.755  0.45223
DonorQX7            0.07021    0.04526   1.551  0.12416
DonorQX8            0.16940    0.04526   3.742  0.00031 ***
DonorQX9            0.44237    0.04526   9.773 4.57e-16 ***
---
Signif. codes:  0 '***' 0.001 '**' 0.01 '*' 0.05 '.' 0.1 ' ' 1

Residual standard error: 0.08468 on 96 degrees of freedom
Multiple R-squared:  0.9994,     Adjusted R-squared:  0.9993
F-statistic:  7262 on 23 and 96 DF,  p-value: < 2.2e-16

Analysis of variance:
Analysis of Variance Table
```

```
Response: log10(Copies)
          Df  Sum Sq Mean Sq   F value    Pr(>F)
Ref.Gene   7 1190.56 170.079 23718.697 < 2.2e-16 ***
Donor     16    7.09   0.443    61.814 < 2.2e-16 ***
Residuals 96    0.69   0.007
---
Signif. codes:  0 '***' 0.001 '**' 0.01 '*' 0.05 '.' 0.1 ' ' 1

Tukey HSD:
  Tukey multiple comparisons of means
    95% family-wise confidence level

Fit: aov(formula = log10(Copies) ~ Ref.Gene + Donor, data = exp22)

$Ref.Gene
                          diff         lwr           upr     p adj
ERV3-AluJ         -0.436843187 -0.52432018 -3.493662e-01 0.0000000
GAPDH-AluJ        -0.139367730 -0.22684473 -5.189073e-02 0.0001169
NAGK-AluJ         -0.137272422 -0.22474942 -4.979543e-02 0.0001555
RPPH1-AluJ        -0.226896921 -0.31437392 -1.394199e-01 0.0000000
TERT-AluJ         -0.012487044 -0.09996404  7.498995e-02 0.9994945
ValidPrime-AluJ   -0.388182134 -0.47565913 -3.007051e-01 0.0000000
GAPDH-ERV3         0.297475457  0.20999846  3.849525e-01 0.0000000
NAGK-ERV3          0.299570765  0.21209377  3.870478e-01 0.0000000
RPPH1-ERV3         0.209946266  0.12246927  2.974233e-01 0.0000000
TERT-ERV3          0.424356143  0.33687915  5.118331e-01 0.0000000
ValidPrime-ERV3    0.048661053 -0.03881594  1.361380e-01 0.6339257
NAGK-GAPDH         0.002095308 -0.08538169  8.957230e-02 1.0000000
RPPH1-GAPDH       -0.087529191 -0.17500619 -5.219554e-05 0.0497604
TERT-GAPDH         0.126880686  0.03940369  2.143577e-01 0.0006127
ValidPrime-GAPDH  -0.248814404 -0.33629140 -1.613374e-01 0.0000000
RPPH1-NAGK        -0.089624499 -0.17710149 -2.147504e-03 0.0409266
TERT-NAGK          0.124785378  0.03730838  2.122624e-01 0.0008006
ValidPrime-NAGK   -0.250909712 -0.33838671 -1.634327e-01 0.0000000
TERT-RNaseP        0.214409877  0.12693288  3.018869e-01 0.0000000
ValidPrime-RNaseP -0.161285213 -0.24876221 -7.380822e-02 0.0000051
ValidPrime-TERT   -0.375695090 -0.46317208 -2.882181e-01 0.0000000
```

Experiment 25

```
Summary:
Call:
lm(formula = log10(Copies) ~ Ref.Gene + Donor + Run - 1, data = exp25)

Residuals:
     Min       1Q   Median       3Q      Max 
-0.33372 -0.05586  0.00699  0.06867  0.20622 

Coefficients:
               Estimate Std. Error t value Pr(>|t|)    
Ref.GeneAluJ    3.32601    0.03630  91.616  < 2e-16 ***
Ref.GeneERV3    2.91469    0.03630  80.286  < 2e-16 ***
Ref.GeneRNaseP  3.12760    0.03630  86.150  < 2e-16 ***
Ref.GeneTERT    3.27795    0.03630  90.292  < 2e-16 ***
DonorQX10      -0.25318    0.04410  -5.741 6.66e-08 ***
DonorQX11      -0.20816    0.04310  -4.830 3.89e-06 ***
DonorQX12      -0.16510    0.04310  -3.831  0.00020 ***
DonorQX14      -0.25001    0.04310  -5.801 5.01e-08 ***
DonorQX15      -0.13927    0.04310  -3.232  0.00157 ** 
DonorQX16      -0.26688    0.04310  -6.193 7.70e-09 ***
DonorQX17      -0.12060    0.04310  -2.798  0.00594 ** 
DonorQX3       -0.27213    0.04310  -6.315 4.24e-09 ***
DonorQX4       -0.20139    0.04310  -4.673 7.50e-06 ***
DonorQX6       -0.06018    0.04310  -1.396  0.16506    
DonorQX7       -0.26643    0.04310  -6.182 8.10e-09 ***
Run2            0.05658    0.02155   2.626  0.00972 ** 
Run3            0.01324    0.02167   0.611  0.54236    
---
Signif. codes:  0 '***' 0.001 '**' 0.01 '*' 0.05 '.' 0.1 ' ' 1

Residual standard error: 0.1056 on 126 degrees of freedom
  (1 observation deleted due to missingness)
Multiple R-squared:  0.9989,     Adjusted R-squared:  0.9988 
F-statistic:  6824 on 17 and 126 DF,  p-value: < 2.2e-16

Analysis of variance:
Analysis of Variance Table

Response: log10(Copies)
           Df  Sum Sq Mean Sq    F value    Pr(>F)    
Ref.Gene    4 1291.53  322.88 28975.2155 < 2.2e-16 ***
Donor      11    1.04    0.09     8.4671 4.893e-11 ***
Run         2    0.08    0.04     3.7668   0.02577 *  
Residuals 126    1.40    0.01
```

```
Signif. codes:  0 '***' 0.001 '**' 0.01 '*' 0.05 '.' 0.1 ' ' 1

Tukey HSD:
  Tukey multiple comparisons of means
    95% family-wise confidence level

Fit: aov(formula = log10(Copies) ~ Ref.Gene + Donor + Run, data = exp25)

$Ref.Gene
                   diff         lwr         upr     p adj
ERV3-AluJ   -0.41131645 -0.47609864 -0.34653426 0.0000000
RNaseP-AluJ -0.19841123 -0.26319342 -0.13362904 0.0000000
TERT-AluJ   -0.04578585 -0.11102913  0.01945743 0.2654870
RNaseP-ERV3  0.21290522  0.14812303  0.27768741 0.0000000
TERT-ERV3    0.36553059  0.30028731  0.43077387 0.0000000
TERT-RNaseP  0.15262537  0.08738209  0.21786865 0.0000001
```

Experiment 32

```
Summary:
Call:
lm(formula = log10(Copies) ~ Ref.Gene + Donor - 1, data = exp32)

Residuals:
      Min        1Q    Median        3Q       Max
-0.255085 -0.041093 -0.005247  0.028915  0.154534

Coefficients:
               Estimate Std. Error t value Pr(>|t|)
Ref.GeneERV3    3.03262    0.04759  63.723  < 2e-16 ***
Ref.GeneRNaseP  3.15455    0.04759  66.285  < 2e-16 ***
Ref.GeneTERT    3.22035    0.04759  67.668  < 2e-16 ***
DonorQX10      -0.18830    0.06366  -2.958 0.005783 **
DonorQX11      -0.14544    0.06366  -2.285 0.029117 *
DonorQX12      -0.06056    0.06366  -0.951 0.348619
DonorQX13       0.07596    0.06366   1.193 0.241597
DonorQX14      -0.22528    0.06366  -3.539 0.001254 **
DonorQX15      -0.05918    0.06366  -0.930 0.359564
DonorQX16      -0.21983    0.06366  -3.453 0.001581 **
DonorQX17       0.01570    0.06366   0.247 0.806779
DonorQX2        0.13336    0.06366   2.095 0.044187 *
DonorQX3       -0.25861    0.06366  -4.062 0.000294 ***
DonorQX4       -0.18050    0.06366  -2.835 0.007870 **
DonorQX5        0.47707    0.06366   7.494 1.57e-08 ***
```

Supplementary Methods Appendix B – Model output (*cont.*)

```
DonorQX6       -0.04491    0.06366  -0.705 0.485667
DonorQX7       -0.23704    0.06366  -3.723 0.000756 ***
DonorQX8       -0.26565    0.06366  -4.173 0.000215 ***
DonorQX9        0.20125    0.06366   3.161 0.003427 **
---
Signif. codes:  0 '***' 0.001 '**' 0.01 '*' 0.05 '.' 0.1 ' ' 1

Residual standard error: 0.07797 on 32 degrees of freedom
Multiple R-squared:  0.9996,     Adjusted R-squared:  0.9994
F-statistic:  4202 on 19 and 32 DF,  p-value: < 2.2e-16

Analysis of variance:
Analysis of Variance Table

Response: log10(Copies)
          Df Sum Sq Mean Sq   F value    Pr(>F)
Ref.Gene   3 483.51 161.170 26511.181 < 2.2e-16 ***
Donor     16   1.88   0.117    19.315 4.635e-12 ***
Residuals 32   0.19   0.006
---
Signif. codes:  0 '***' 0.001 '**' 0.01 '*' 0.05 '.' 0.1 ' ' 1

Tukey HSD:
    Tukey multiple comparisons of means
    95% family-wise confidence level

Fit: aov(formula = log10(Copies) ~ Ref.Gene + Donor, data = exp32)

$Ref.Gene
                  diff          lwr       upr     p adj
RNaseP-ERV3 0.12193033 5.621158e-02 0.1876491 0.0002055
TERT-ERV3   0.18773816 1.220194e-01 0.2534569 0.0000002
TERT-RNaseP 0.06580782 8.907612e-05 0.1315266 0.0496339
```

Supplementary Data

cfDNA load in 12 donors based on 3 reference genes (TERT, RPPH1, ERV-3): mean values and 95% confidence intervals (Figure 7)

*Standard errors are calculated based on ANOVA for each donor

		Natural log scale (copies/mL plasma)									Linear copy number scale (copies/mL plasma)				
	**Donor ID**	**Exp 025a**	**Exp 025b**	**Exp 025c**	**Mean (TERT)**	**Mean (3 reference genes)**	**Standard error***	**95% confidence interval (CI)**	**Upper CI**	**Lower CI**	**Mean (3 reference genes)**	Upper CI	Lower CI	Upper error bar	Lower error bar
TERT	1	7.67	7.63	7.60	7.63	7.24	0.23	1.00	8.24	6.24	1396	3788	515	2392	882
TERT	3	6.93	7.22	7.11	7.09	6.63	0.28	1.18	7.82	5.45	760	2482	233	1722	527
TERT	4	7.16	7.13	7.14	7.14	6.76	0.30	1.28	8.04	5.48	862	3103	239	2241	623
TERT	6	7.42	7.63	7.17	7.41	7.12	0.15	0.65	7.78	6.47	1242	2385	647	1143	595
TERT	7	7.17	7.06	7.11	7.12	6.58	0.31	1.32	7.90	5.27	723	2695	194	1972	529
TERT	10	7.20	6.75		6.97	6.58	0.25	1.08	7.66	5.50	722	2129	245	1406	477
TERT	11	6.77	7.00	7.19	6.99	6.69	0.20	0.84	7.54	5.85	806	1874	346	1068	459
TERT	12	7.42	7.51	7.16	7.36	6.77	0.31	1.32	8.09	5.45	871	3250	233	2379	638
TERT	14	6.57	6.96	7.05	6.86	6.67	0.23	0.99	7.66	5.69	792	2121	296	1329	496
TERT	15	7.18	7.21	7.31	7.24	6.89	0.21	0.92	7.81	5.97	982	2453	393	1471	589
TERT	16	6.97	7.14	7.15	7.09	6.58	0.26	1.13	7.71	5.45	718	2226	232	1507	486
TERT	17	6.98	7.23	7.54	7.25	6.88	0.27	1.15	8.03	5.74	975	3073	310	2097	666

Average variation due to Reference gene 0.25

	**Donor ID**	**Exp 025a**	**Exp 025b**	**Exp 025c**	**Mean (RNase P)**
RNase P	1	6.99	7.56	7.24	7.26
RNase P	3	6.88	7.18	5.98	6.68
RNase P	4	7.02	6.97	6.90	6.96
RNase P	6	7.09	7.07	7.08	7.08
RNase P	7	6.50	6.68	6.56	6.58
RNase P	10	6.59	7.09	6.45	6.71
RNase P	11	6.75	7.01	6.55	6.77
RNase P	12	6.47	6.92	6.44	6.61
RNase P	14	7.06	7.07	6.71	6.95
RNase P	15	6.79	7.04	6.97	6.93
RNase P	16	6.46	6.60	6.24	6.43
RNase P	17	6.76	7.43	6.91	7.03

Supplementary Data (cont.)

	Donor ID	Exp 025a	Exp 025b	Exp 025c	Mean (ERV-3)
ERV-3	1	6.76	7.03	6.70	6.83
	3	5.81	6.52	6.08	6.14
	4	6.48	6.12	5.92	6.17
	6	6.72	6.89	7.05	6.89
	7	6.00	5.92	6.24	6.06
	10	6.22	6.30	5.68	6.07
	11	6.18	6.57	6.21	6.32
	12	6.57	6.09	6.36	6.34
	14	6.47	5.72	6.47	6.22
	15	6.57	6.27	6.66	6.50
	16	6.50	6.51	5.62	6.21
	17	5.85	6.33	6.91	6.37
